# Design, modeling, and performance analysis of a 2-DOF pneumatic flexible wrist with controllable braking torque

**DOI:** 10.1371/journal.pone.0362364

**Published:** 2026-11-11

**Authors:** Hongbo Liu, Shuo Wang, Wenpeng Xu

**Affiliations:** 1 Engineering Training Center, Beihua University, Jilin, Jilin, China; 2 School of Mechanical Engineering, Beihua University, Jilin, Jilin, China; Hubei Polytechnic University, CHINA

## Abstract

To address the structural complexity and limited compliance of traditional rigid robotic wrists, as well as the absence of adjustable stiffness in soft wrists designed primarily for flexibility, a two-degree-of-freedom (2-DOF) pneumatic flexible wrist with controllable braking torque was developed. The wrist comprises three circumferentially arranged pneumatic actuators connected in parallel around a central pneumatic cross-hinge brake, enabling multidirectional pivoting, while the braking torque can be adjusted according to task requirements for pose stabilization. This configuration combines the high stiffness required for posture maintenance during braking with compliant actuation during motion. Theoretical models of the wrist pivot angle, pivoting direction, and braking torque were established. The pivot-angle model was empirically calibrated using the measured pivot-angle data, while the pivoting-direction and braking-torque models were validated experimentally. Mechanical performance was evaluated under constant-friction-torque and constant-pivot-angle conditions, and empirical relations were obtained by fitting the experimental data. A three-dimensional motion-capture system was also used to characterize the dynamic response under different braking conditions and excitation signals. Under the present experimental conditions, the results show that the wrist exhibits high compliance and enables continuous omnidirectional pivoting with a maximum single-axis pivot angle of 45.2°. The pneumatic cross‑hinge brake achieves multidirectional braking with continuously adjustable torque, reaching a maximum braking torque of 0.842 N·m under brake-only actuation at 0.35 MPa. During coordinated actuation–braking operation, the wrist’s overall resistance to external loads increased by a factor of 4.98 compared with its unpressurized initial state. This composite load-resisting capacity originates from the combined effects of the pneumatic actuators and the central brake.

## Introduction

The wrist is a key component between the robotic arm and the end-effector. It adjusts the spatial pose of the end-effector and establishes controlled contact with the target object. The structural characteristics and motion performance of the wrist directly affect the positioning accuracy of the end-effector and, consequently, the stability and precision of the overall operation [1,2]. Conventional rigid robotic wrists are generally made of metal and actuated by motors [3,4], hydraulics [5,6], or rotary pneumatic cylinders [7]. These wrists offer high load capacity and positioning accuracy, rapid response, and relatively straightforward modeling and control. However, in unstructured environments or during interaction with compliant workpieces and humans, their high rigidity and limited cushioning may scratch workpieces or cause structural damage. The size and mass of rigid linkages also increase the load on the robotic arm and impair system dynamics. During human-robot collaboration, a collision may additionally cause injury [8,9]. Therefore, flexible wrists fabricated from low-modulus soft materials have attracted extensive attention in recent years due to their advantages in active and passive adaptability, safety, and compatibility in human-robot and machine-environment interactions [10,11]. Flexible wrists generally use three actuation modes: pneumatic actuation [12,13], tendon or cable actuation [14–16], and smart-material actuation, including shape memory alloys (SMA), ionic polymer-metal composites (IPMC), and twisted and coiled polymers (TCP/TCA) [17–21]. Among these approaches, pneumatic actuation offers a simple structure, low weight, and high compliance. Applied in wrist design, pneumatic actuation can improve interaction safety and operational dexterity. For instance, Andrikopoulos et al. [22] developed a 2-DOF wrist exoskeleton using four FESTO pneumatic muscles. A rigid ABS frame constrains actuator deformation, while a nonlinear PID control algorithm is used to achieve trajectory tracking. Although the device is structurally simple and inexpensive, its rigid frame and high-pressure air supply limit wearability and portability. Lai et al. [23] proposed a soft wrist rehabilitation robot employing double-layer lattice pneumatic actuators (DLSPAs). After model-based parameter optimization, the dorsiflexion and palmar-flexion assistance forces reached 4.85 and 5.11 N at angles of 36.40° and 38.36°, respectively. This represented a 156% increase in output force over the single-layer structure. Despite the improved output, the structural complexity, dependence on an external air source, and potential fatigue-life limitations remain to be addressed. Bartlett et al. [24] presented a 2-DOF wrist rehabilitation device driven by four McKibben muscles, enabling flexion-extension and rotation through coordinated contraction. Although compact, the device places the muscles in direct contact with the skin, posing a safety risk in the event of actuator failure. Al-Fahaam et al. [25] designed a wrist orthosis actuated by modified bending and contracting pneumatic muscles to realize flexion-extension and abduction-adduction. However, strong nonlinear coupling complicates high-precision modeling and control. Liu et al. [26] developed a multifunctional soft manipulator integrating fingers and a wrist. The wrist used a hollow cylindrical structure divided into four equal sectors and incorporates a conformal natural rubber core and a centrally embedded inextensible fiber to form four sealed chambers. At 0.20 MPa, the wrist achieved a maximum bending angle of 90.8°, with adjustable bending direction and angle via pneumatic control, enabling multidirectional grasping. Guan et al. [27] proposed a flexible humanoid manipulator with an integrated multi-bending wrist actuated symmetrically by four BC-PAMs, enabling multidirectional bending up to 30°. This design combined high load capacity with posture adjustment, improving adaptability in complex tasks. Yao et al. [28] developed an enhanced pneumatic actuator (RSPA) incorporating a TPU airbag with a composite nylon reinforcement layer and an elastic support structure, and integrated it into a soft wearable upper-limb rehabilitation robot. The robot’s wrist consists of four RSPAs. Independently controlling the air-supply configuration and pressure of each RSPA enables 2-DOF motion training and adaptive adjustment of the motion intensity. Overall, existing pneumatically actuated flexible wrists make effective use of the compliance and adaptability of soft materials but generally lack active stiffness adjustment. They therefore struggle to maintain the end-effector pose under varying external loads.

To improve the ability of flexible wrists to withstand varying external loads, tunable-stiffness and damping mechanisms have been introduced to increase load capacity and motion accuracy. Hu et al. [29] developed a biomimetic flexible wrist that provides 3-DOF motion and variable stiffness through a compact particle-jamming joint (PJJ) and six lightweight soft-ring-reinforced bellows pneumatic actuators (SRBPA). The wrist demonstrated an axial load capacity exceeding 60 N, a torque exceeding 1.8 N·m, and minimal radial expansion of 3.7%, thereby combining high load capacity with structural stability. However, the PJJ increases control complexity, while the SRBPA requires high manufacturing precision and increases system cost. Liu et al. [30] proposed a variable-stiffness actuator based on composite fiber-particle jamming and established a corresponding mechanical model. Experiments showed that the actuator achieved a maximum stiffness of 0.1825 N/mm, representing improvements of 88.05% and 26.73% over the pure-fiber and pure-particle structures, respectively. Its stability also increased by 67.43%, confirming the overall advantage of the composite jamming mechanism. The VarWrist system integrates bellows actuation with a fiber jamming variable stiffness mechanism [31]. The wrist’s range of motion (flexion/extension 81.9°/78.5°, ulnar/radial deviation 70.5°) and trajectory characteristics (similarity improvement of 73%) closely matched those of the human wrist. Its stiffness adjustment ratios at rest and during operation reached 206% and 155%, respectively, indicating a balance between load capacity and control precision. In pneumatic soft robotics, particle-jamming variable-stiffness mechanisms are widely used because of their simple structure, broad adjustment range, and rapid response. However, the need for external vacuum pumps and tubing reduces portability, while stiffness switching is delayed and vibration resistance remains limited in the jammed state. Antagonistic actuation uses two or more oppositely acting wrist actuators to simultaneously provide motion and stiffness modulation and is widely applied in compliant robotic systems [32–34]. However, stiffness adjustment is delayed and nonlinear, impact-load tolerance is limited, and motion-stiffness decoupling requires complex control.

To overcome the bulky structure, slow response, and limited stability of granular-jamming variable-stiffness systems, as well as the structural redundancy, energy consumption, restricted adjustment range, and coupled vibration of antagonistic systems, this study introduces a co-actuation design that combines pneumatic actuators with a pneumatic brake. The design is intended to improve load adaptability and posture-maintenance capability under varying external loading conditions. Based on this concept, a 2-DOF pneumatic flexible wrist with controllable braking torque was developed. The proposed wrist combines compliant actuation with high-stiffness braking in a compact architecture, enabling dexterous 2-DOF motion and greater adaptability to load variations. Mathematical modeling, experimental evaluation, and dynamic characterization were performed to assess the design. The results demonstrate that the wrist coordinates compliant actuation during motion with high-stiffness posture maintenance during braking, thereby preserving compliant interaction while providing stable load support. The proposed architecture provides a practical approach for improving the performance of pneumatic flexible wrists in load-varying applications.

## Structure and function of the flexible wrist

### Structural composition of the wrist

The proposed wrist consists of three circumferentially arranged pneumatic actuators and a centrally mounted pneumatic cross-hinge brake in parallel, providing the wrist with two degrees of freedom. By regulating the pressurization patterns and pressure differentials among the actuators, the wrist can perform human-like pitch, yaw, and coupled motions, as shown in Fig 1A.

**Fig 1:**
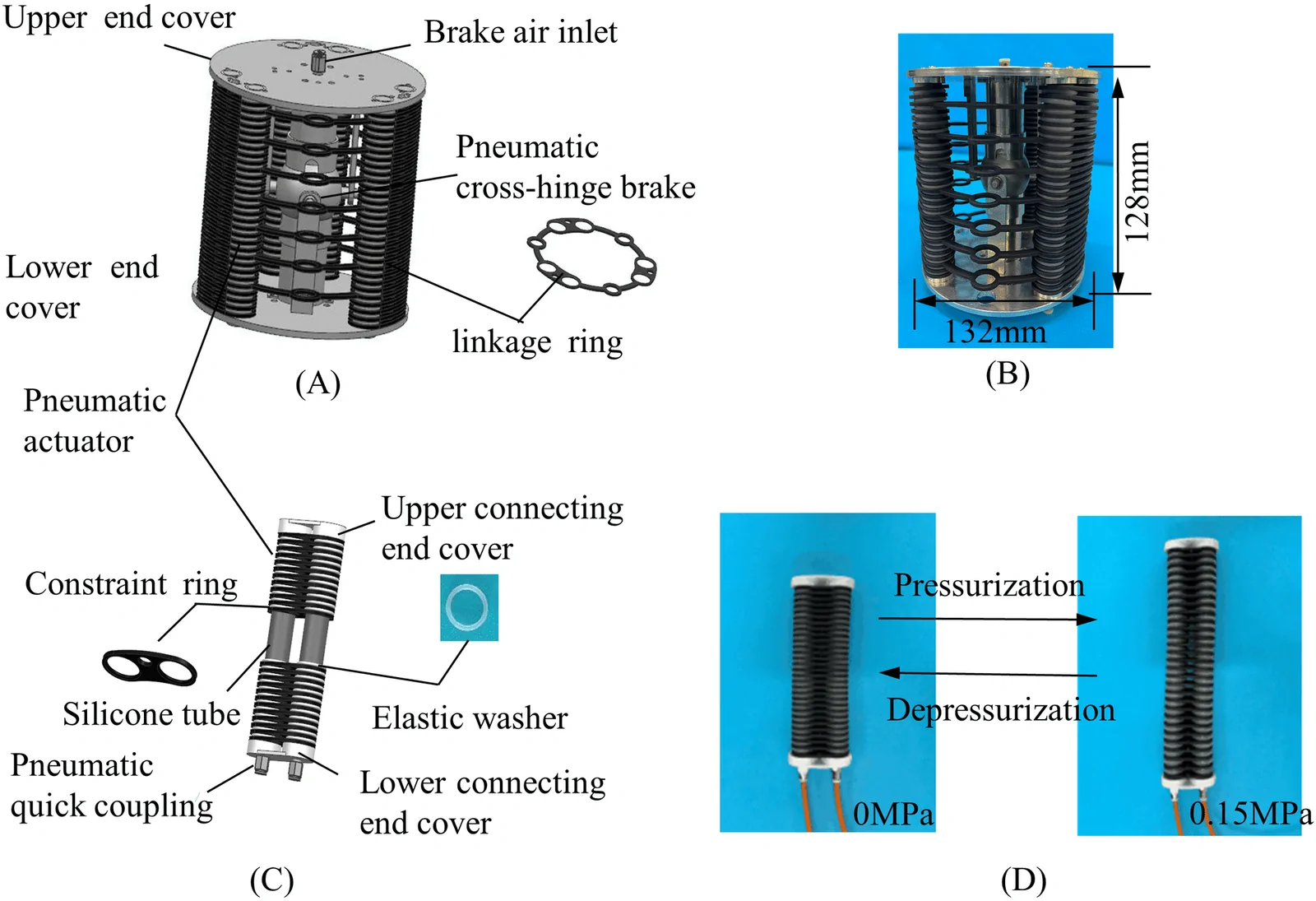
Pneumatic flexible wrist. (A) 3D model. (B) Prototype. (C) Actuator model. (D) Actuator elongation.

As shown in Fig 1B, the wrist body is cylindrical with an initial height of 128 mm and a radial dimension of 132 mm. Three pneumatic actuator groups are located at a radial distance of 55 mm and uniformly spaced at 120° intervals around the circumference. Each actuator consists of two parallel extension-type pneumatic artificial muscles [2] to increase its driving capacity. To maintain the radial positions of the three actuator groups and ensure coordinated deformation, seven linkage rings are placed at 15-mm intervals along the axial direction. Except for the elastic airbags, constraint rings, elastic washers, and linkage rings in the actuators and brake, the remaining structural components are made of aluminum alloy. The assembled wrist weighs 738 g.

As shown in Fig 1C, the pneumatic actuator comprises upper and lower connecting end covers, silicone tubes, constraint rings, elastic washers, and plugs. To suppress radial expansion of the silicone tube, identical constraint rings are fitted around its outer surface, with elastic washers inserted between adjacent rings. The elastic washers are made of VMQ silicone. These washers restrict radial expansion and moderately increase actuator deformation under compression, thereby enlarging the wrist pivot angle. When the two artificial muscles are supplied with compressed air at a constant pressure, the radial expansion of the silicone tube is restricted by the constraint rings and elastic washers. The resulting axial force extends the actuator, and its elongation increases with air pressure. After depressurization, the actuator returns to the initial state due to the intrinsic elasticity of the silicone tube, as shown in Fig 1D.

### Structure and function of the pneumatic cross-hinge brake

The pneumatic cross-hinge brake consists mainly of upper and lower end covers, braking airbags, friction plates, braking balls, sleeves, and lug plates. The pneumatic cross-hinge brake engages under pressure and permits free rotation after depressurization, as shown in Fig 2A. The upper and lower end covers are identical in structure and size, and their end faces contain mounting holes for the lugs, sleeve, and pneumatic connectors. The lugs on the upper and lower end covers have identical structures and are installed with a 90° phase difference. The bottom face of each lug plate contains a threaded hole for bolt connection with the end cover. A countersunk through-hole at the other end accommodates a deep-groove ball bearing. A braking airbag is mounted inside the sleeve, and both ends of the airbag are sealed with upper and lower plugs to form a sealed cavity. The upper plug plate contains a threaded hole for bolted connection to the friction plate, while the lower plug plate contains a threaded hole that connects to the pneumatic connector. A thin-walled braking ball with the same curvature as the two friction plates is installed between them, with its center 60 mm from both end covers. Four stepped shafts are uniformly distributed around the equator of the braking ball. Each shaft forms an interference fit with the bearing at the end of the corresponding lug. The four lug plates are uniformly distributed on a 54-mm circumference. Fig 2B shows the brake prototype, which weighs 460 g.

**Fig 2:**
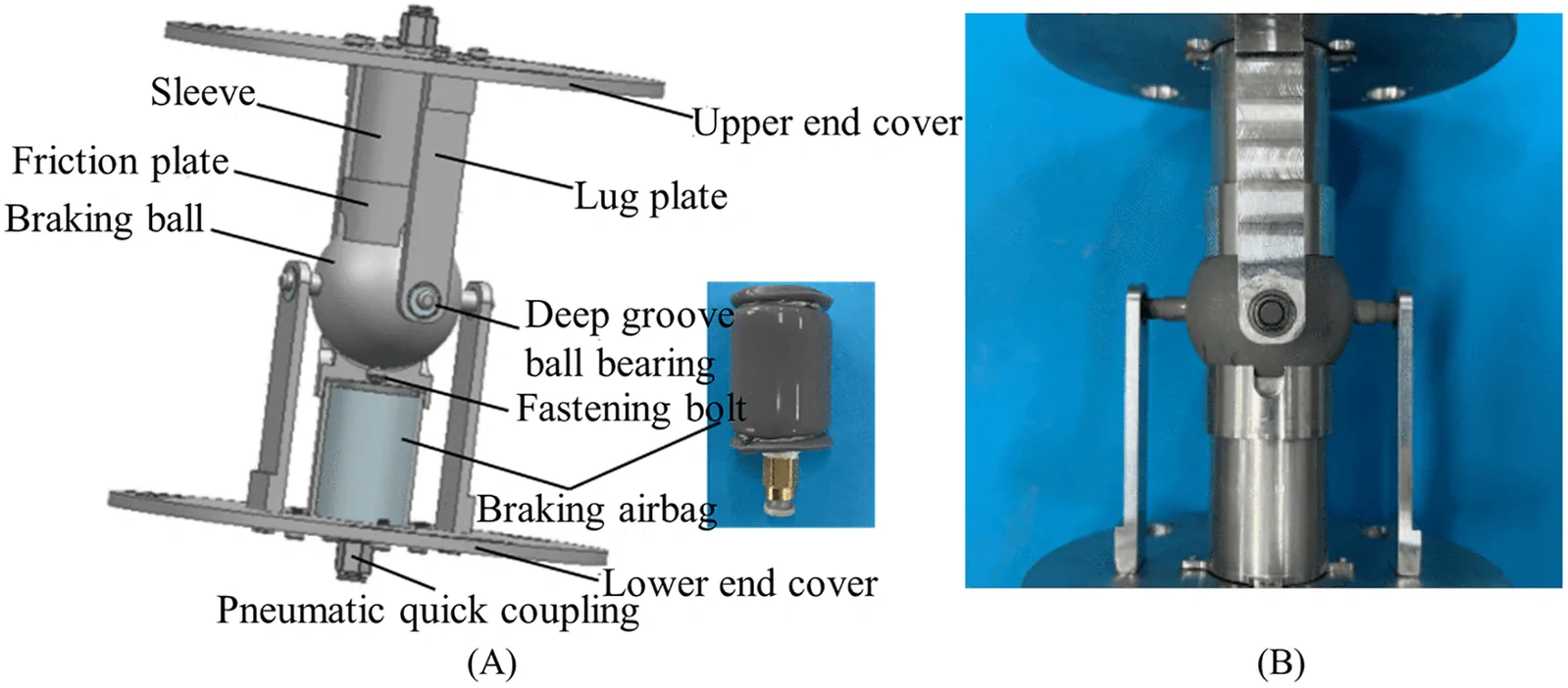
Pneumatic cross-hinge brake. (A) 3D model. (B) Prototype.

When compressed air is introduced into the braking airbag, the airbag expands. The sleeve restricts radial expansion, so the airbag elongates axially and pushes the friction plates toward the braking ball. As the pressure increases, the friction plates contact the braking ball, generating a normal force. The resulting friction torque resists relative motion and produces the braking action. Braking capacity increases with airbag pressure. When the air pressure decreases, the braking airbag resets under its own elastic force, and the friction force between the friction plates and the braking ball decreases to zero, releasing the brake. Therefore, the magnitude and direction of the braking torque can be controlled by adjusting the pressures in the two braking airbags to accommodate different load conditions.

### Working principle of the wrist

When compressed air is supplied, the inner wall of the silicone tube in the actuator expands under pressure. The outer constraint rings and elastic washers restrict radial expansion, thereby generating an axial driving force. Because the central brake is axially inextensible, it constrains axial elongation of the wrist, allowing only bending deformation. When the actuator is depressurized, the elasticity of the silicone tube returns the wrist to its initial position. Owing to the wrist’s structural symmetry, different pressures applied to the three actuator groups produce multidirectional pivoting about the brake center, as shown in Fig 3. Once the wrist reaches the target position, the pressure in the corresponding braking airbag is adjusted to engage the brake and hold the posture. Therefore, through coordinated actuator-brake operation, the pivoting direction, pivot angle, and output force of the wrist can be adjusted to suit different task requirements, while the braking torque can be adjusted according to task requirements for pose stabilization.

**Fig 3:**
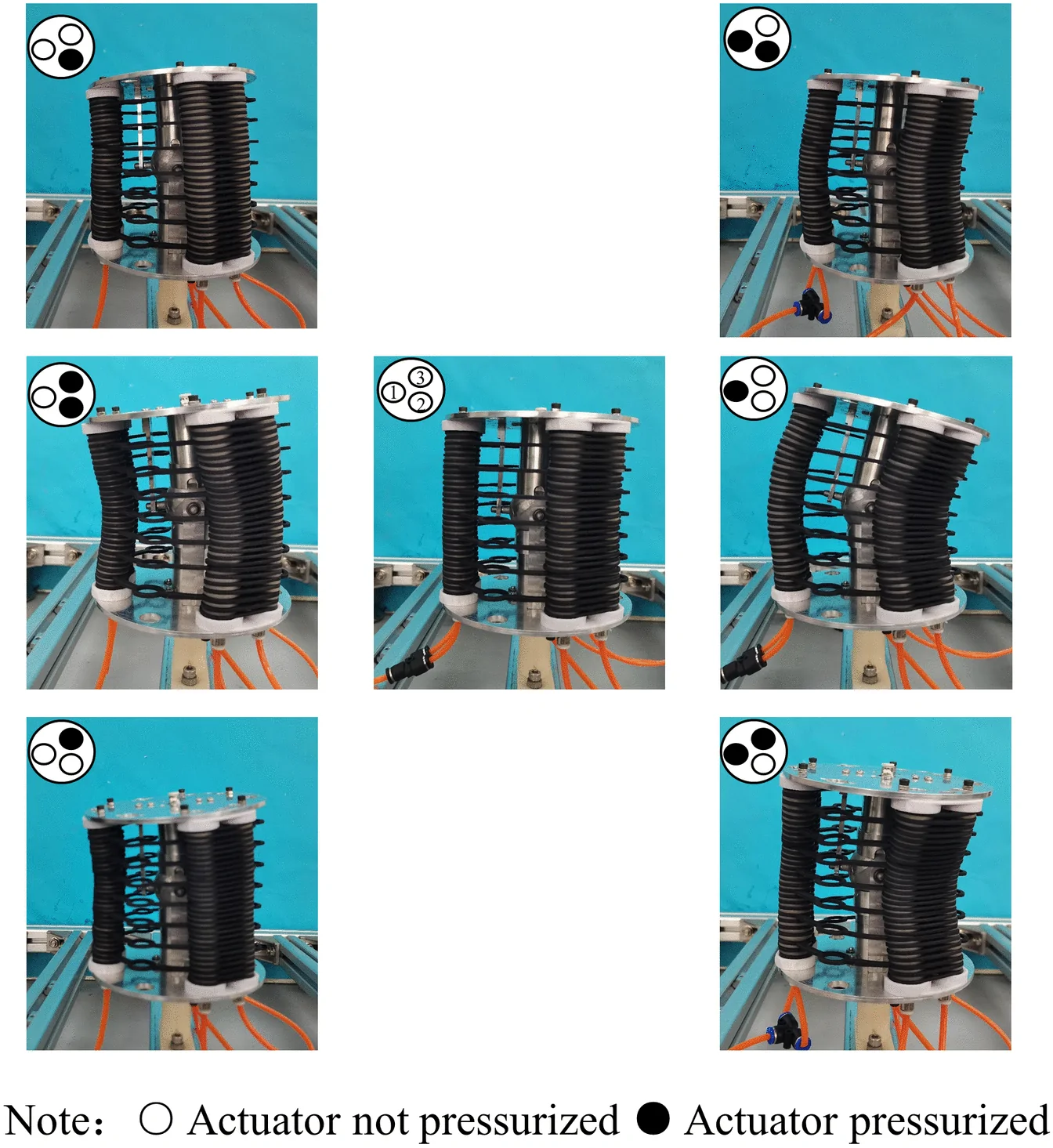
Multidirectional wrist pivot posture.

## Static modeling of the wrist

When the three actuators are supplied with compressed air at different pneumatic pressures, the upper end cover of the wrist pivots about the brake center in the direction of the resultant actuator torque. The direction of the resultant-torque vector determines the pivoting direction, whereas its magnitude determines the pivot angle.

The torque analysis of the upper end cover in Fig 4B identifies four components: the driving torque generated by the actuator is $M_{p}$, the impedance torque generated by the deformation of the silicone tube inside the actuator is $M_{g}$, the impedance torque generated by the compression of the elastic washer is $M_{t}$, and the friction torque generated by relative rotation between the braking ball and the lug plate is $M_{f}$.

**Fig 4:**
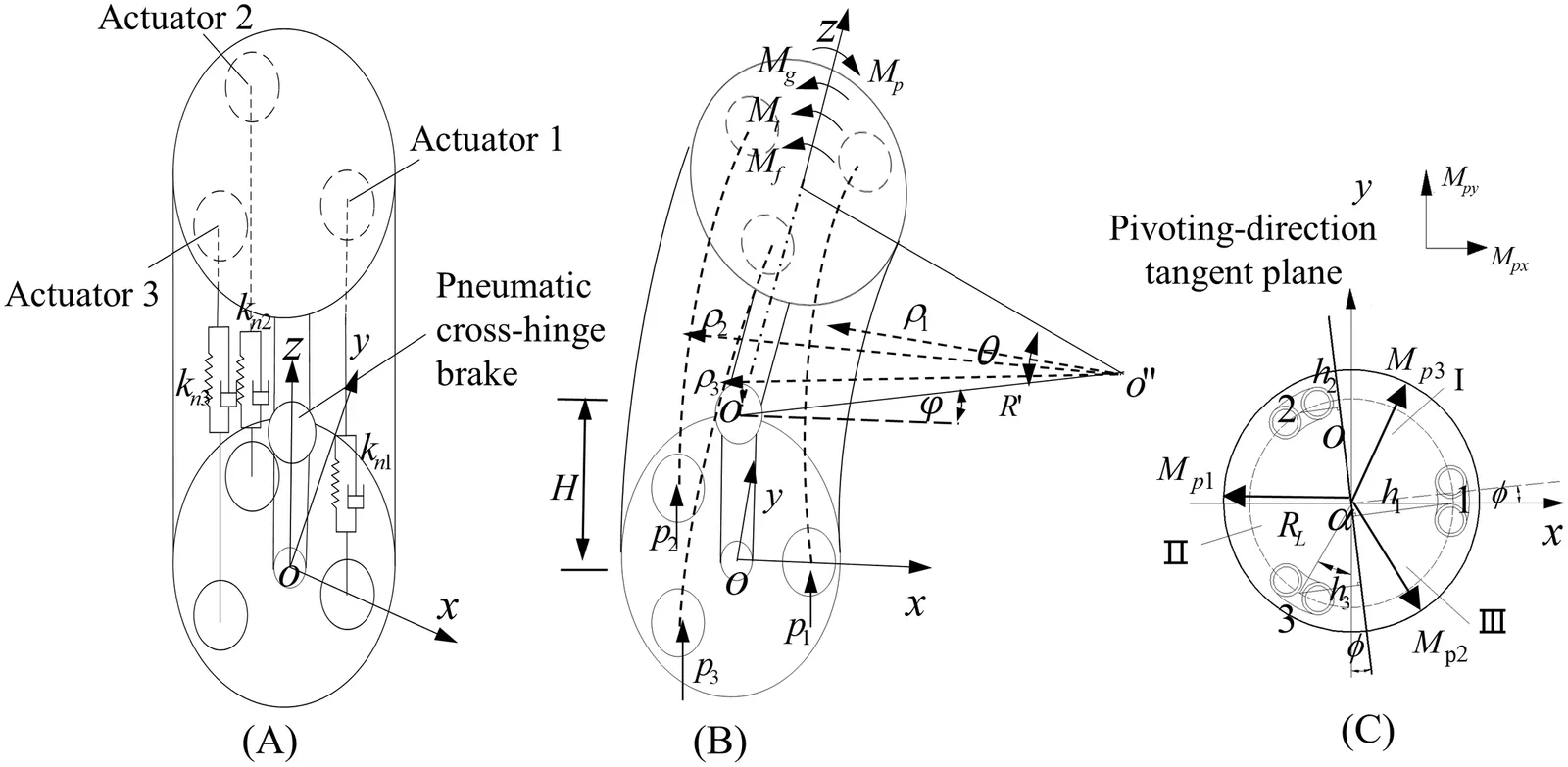
Torque analysis of the wrist. (A) Initial state. (B) Multidirectional pivoting under pneumatic pressure. (C) Pivoting direction analysis.

The driving torque applied to the upper end cover of the wrist when compressed air is supplied is:


$$\begin{array}{c} \begin{array}{c} M_{p}=\sum_{i=\text{1}}^{\text{3}}M_{pi} \end{array} \end{array}$$ (1)

The driving torque generated by a single actuator is:


$$\begin{array}{c} \begin{array}{c} M_{pi}=\text{2}p_{i}S_{i}R_{L} \end{array} \end{array}$$ (2)

where $p_{i}$ denotes the air pressure applied to actuator i, $S_{i}$ represents the cross-sectional area of the internal chamber of the artificial muscle [35], and $R_{L}$ is the distance from the actuator center to the wrist center.

### Theoretical model of wrist pivoting direction angle

The wrist pivoting direction is determined primarily by the orientation of the actuation-torque vector. As shown in Fig 4C, the torque analysis of the upper end cover gives:


$$\begin{array}{c} \begin{array}{c} \varphi =arctan\frac{M_{py}}{M_{px}} \end{array} \end{array}$$ (3)

$$\begin{array}{c} \begin{array}{c} M_{px}=\left(M_{p\text{2}}+M_{p\text{3}}\right)sin\alpha -M_{p\text{1}} \end{array} \end{array}$$ (4)

$$\begin{array}{c} \begin{array}{c} M_{py}=\left(M_{p\text{3}}-M_{p\text{2}}\right)cos\alpha \end{array} \end{array}$$ (5)

where φ represents the wrist pivoting direction angle; $M_{px}$ denotes the resultant-torque component in the x -direction; $M_{py}$ represents the resultant-torque component in the y -direction; α is the angle between the resultant-torque vector and the y -axis, which can be calculated geometrically as $\alpha =\frac{\pi }{\text{6}}$.

Substituting Eqs. (4) and (5) into Eq. (3) yields the relationship between the wrist pivoting direction and the air pressure applied to each actuator as follows:


$$\begin{array}{c} \begin{array}{c} \varphi =arctan\frac{\sqrt{\text{3}}\left(p_{\text{3}}S_{\text{3}}-p_{\text{2}}S_{\text{2}}\right)}{p_{\text{2}}S_{\text{2}}+p_{\text{3}}S_{\text{3}}-\text{2}p_{\text{1}}S_{\text{1}}} \end{array} \end{array}$$ (6)

### Theoretical model of wrist pivot angle

When compressed air is supplied to the three actuators, the driving torque causes the wrist to pivot freely in different directions about the brake center. The torques resisting wrist motion are $M_{g}$, $M_{t}$, and $M_{f}$, respectively. Torque equilibrium at the upper end cover gives:


$$\begin{array}{c} \begin{array}{c} \sum_{i=\text{1}}^{\text{3}}M_{pi}=\sum_{i=\text{1}}^{\text{3}}M_{gi}+\sum_{i=\text{1}}^{\text{3}}M_{ti}+\sum_{i=\text{1}}^{\text{4}}M_{fi} \end{array} \end{array}$$ (7)

The actuators are uniformly distributed at 120° intervals on a circle of radius $R_{L}$. Owing to structural symmetry, the pivoting behavior is identical in the three equally divided regions, denoted I, II, and III. For Region I, the actuator deformation states for pivoting direction angles between 0 and 2π/3 are:

① When 0 ≤ *ϕ* ≤ π/6, actuators 2 and 3 extend, while actuator 1 contracts.
② When π/6 < *ϕ* ≤ π/2, actuator 3 extends, while actuators 1 and 2 contract.
③ When π/2 < *ϕ* ≤ 2π/3, actuators 1 and 2 extend, while actuator 3 contracts.

#### Torque analysis of deformation case ①.

In this case, actuators 2 and 3 elongate, whereas actuator 1 compresses, as illustrated in Fig 4B. For simplicity, each deformed actuator is assumed to follow a circular arc. The wrist pivot angle is denoted by θ, and the distance from the braking-ball center to the lower end cover by H.

During deformation, the arc length and radius of curvature of each actuator can be expressed as follows:


$$\begin{array}{c} \begin{array}{c} \left\{ \begin{array}{cc} & R^{\prime }=Hcot\theta \\ & \rho_{\text{1}}=(Hcot\theta -h_{\text{1}})\theta \\ & \rho_{\text{2}}=(Hcot\theta +h_{\text{2}})\theta \\ & \rho_{\text{3}}=(Hcot\theta +h_{\text{3}})\theta \end{array}\right. \end{array} \end{array}$$ (8)

where $R^{\prime }$ denotes the radius of curvature of the wrist central axis, corresponding to line segment $O^{\prime }O"$; $\rho_{\text{1}}$, $\rho_{\text{2}}$, and $\rho_{\text{3}}$ denote the radii of curvature of the centerlines of actuators 1, 2, and 3, respectively; $h_{\text{1}}$, $h_{\text{2}}$, and $h_{\text{3}}$ denote the distances from the centers of actuators 1, 2, and 3 to the tangent plane of the wrist during pivoting, respectively. From the geometric relationship shown in Fig 4C:


$$\begin{array}{c} \begin{array}{c} \left\{ \begin{array}{cc} & h_{\text{1}}=R_{L}sin(\pi /\text{2}-\varphi )\\ & h_{\text{2}}=R_{L}sin(\alpha -\varphi )\\ & h_{\text{3}}=R_{L}sin(\alpha +\varphi ) \end{array}\right. \end{array} \end{array}$$ (9)

Using the arc-length relation and Eq. (9), the deformation compatibility equations of each actuator after wrist deformation are expressed as follows:


$$\begin{array}{c} \begin{array}{c} \left\{ \begin{array}{cc} & \Delta l_{\text{1}}=H-\rho_{\text{1}}\theta \\ & \Delta l_{\text{2}}=\rho_{\text{2}}\theta -H\\ & \Delta l_{\text{3}}=\rho_{\text{3}}\theta -H \end{array}\right. \end{array} \end{array}$$ (10)

where $\Delta l_{\text{1}}$, $\Delta l_{\text{2}}$, and $\Delta l_{\text{3}}$ denote the changes in the centerline arc lengths of actuators 1, 2, and 3, respectively.

The driving torque produced by the air pressure acting on the wrist’s upper end cover is given by:


$$\begin{array}{c} \begin{array}{c} M_{p}=\sum_{i=\text{1}}^{\text{3}}M_{pi}=\text{2}p_{\text{2}}S_{\text{2}}h_{\text{2}}+\text{2}p_{\text{3}}S_{\text{3}}h_{\text{3}}-\text{2}p_{\text{1}}S_{\text{1}}h_{\text{1}} \end{array} \end{array}$$ (11)

During wrist pivoting, the impedance torque generated by the silicone tube comprises two components: the torque $M_{n}$ caused by the bending deformation of the silicone tube around the main body, and the coupling torque $M_{r}$ associated with axial stiffness.

Assuming that the impedance torques generated by the silicone tube are linearly superposed, the resultant torque $M_{g}$ can be expressed as:


$$\begin{array}{c} \begin{array}{c} \sum_{i=\text{1}}^{\text{3}}M_{gi}=\text{2}M_{n\text{1}}+\text{2}M_{n\text{2}}+\text{2}M_{n\text{3}}+\text{2}k_{n}\left(\Delta l_{\text{2}}h_{\text{2}}+\Delta l_{\text{3}}h_{\text{3}}-\Delta l_{\text{1}}h_{\text{1}}\right) \end{array} \end{array}$$ (12)

where $M_{n\text{1}}$ $M_{n\text{2}}$, and $M_{n\text{3}}\;$ denote the resistance torques caused by the bending deformation of the silicone tube body in actuators 1, 2, and 3, respectively.

The relationship between the bending-resistance torque of the silicone tube body and the bending angle is given by [35]:


$$\begin{array}{c} M_{n}=\frac{\pi E(D_{\text{1}}^{\text{2}}-D_{\text{2}}^{\text{2}})\left[\left(D_{\text{1}}^{\text{2}}+D_{\text{2}}^{\text{2}}\right)l_{\text{0}}^{\text{2}}+\text{2}D_{\text{1}}^{\text{2}}l_{\text{0}}\Delta l\right]}{\text{6}\text{4}(l_{\text{0}}+\Delta l{)}^{\text{3}}}\theta ;k_{n}=\frac{\pi E(D_{\text{1}}^{\text{2}}-D_{\text{2}}^{\text{2}})l_{\text{0}}(l_{\text{0}}-\Delta l^{\text{2}})}{\text{4}(l_{\text{0}}+\Delta l{)}^{\text{4}}} \end{array}$$

where $k_{n}$ is the axial stiffness of the silicone tube.

For actuator 1, the elastic washer undergoes compression during wrist motion and generates an impedance torque that opposes deformation. The elastic washer is therefore modeled as an equivalent spring. Similar to the silicone tube, its impedance torque includes the torque caused by compression and the coupling torque associated with axial stiffness. Because each elastic washer is thin and undergoes only a small portion of the total pivot angle, the impedance torque resulting from its own compressive deformation is neglected, and only the axial-stiffness coupling torque is considered. Therefore, the impedance torque generated by the elastic washer in actuator 1 can be expressed as:


$$\begin{array}{c} \begin{array}{c} M_{t\text{1}}=\frac{\text{2}k_{t}\Delta l_{\text{1}}h_{\text{1}}}{N} \end{array} \end{array}$$ (13)

where $k_{t}$ is the axial stiffness of the elastic washer, and N is the number of elastic washers in the actuator.

The axial forces generated when different air pressures are applied to the three actuators are expressed as follows:


$$\begin{array}{c} \begin{array}{c} \sum_{i=\text{1}}^{\text{3}}F_{pi}=\text{2}p_{\text{2}}S_{\text{2}}+\text{2}p_{\text{3}}S_{\text{3}}-\text{2}p_{\text{1}}S_{\text{1}} \end{array} \end{array}$$ (14)

The brake prevents axial extension of the wrist. The axial force generated by the actuator under pneumatic pressure is transmitted to the bearing at the interface between the braking ball and the lug plate, thereby generating a frictional torque.


$$\begin{array}{c} \begin{array}{c} \sum_{i=\text{1}}^{\text{4}}M_{fi}=\mu d_{\text{0}}\sum_{i=\text{1}}^{\text{3}}F_{pi} \end{array} \end{array}$$ (15)

The bearing friction coefficient μ depends on the bearing type, external load, rotational speed, and lubrication condition. Under steady operation, the friction coefficient of a deep-groove ball bearing is generally 0.001–0.0015. $d_{\text{0}}$ represents the nominal inner diameter of the rolling bearing between the stepped shaft of the braking ball and the lug plate. Owing to the small friction coefficient and the relatively small nominal diameter $d_{\text{0}}$, the corresponding friction torque is approximately three orders of magnitude smaller than the driving torque. The bearing friction torque is therefore neglected in the subsequent model.

#### Torque analysis of deformation case ②.

In this case, actuator 3 undergoes elongation, whereas actuators 1 and 2 undergo compression. The driving torque generated by the air pressure acting on the upper end cover of the wrist is expressed as:


$$\begin{array}{c} \begin{array}{c} \sum_{i=\text{1}}^{\text{3}}M_{pi}=\text{2}p_{\text{3}}S_{\text{3}}h_{\text{3}}-\text{2}p_{\text{1}}S_{\text{1}}h_{\text{1}}-\text{2}p_{\text{2}}S_{\text{2}}h_{\text{2}} \end{array} \end{array}$$ (16)

From the geometric relationship, it follows that:


$$\begin{array}{c} \begin{array}{c} \left\{ \begin{array}{cc} & \rho_{\text{1}}=(Hcot\theta -h_{\text{1}})\theta \\ & \rho_{\text{2}}=(Hcot\theta -h_{\text{2}})\theta \\ & \rho_{\text{3}}=(Hcot\theta +h_{\text{3}})\theta \end{array}\right.\;\left\{ \begin{array}{cc} & h_{\text{1}}=R_{L}sin(\pi /\text{2}-\varphi )\\ & h_{\text{2}}=R_{L}sin(\varphi -\alpha )\\ & h_{\text{3}}=R_{L}sin(\varphi +\alpha ) \end{array}\right. \end{array} \end{array}$$ (17)

Within this pivoting range, the deformation compatibility relations among the actuators are expressed as:


$$\begin{array}{c} \begin{array}{c} \left\{ \begin{array}{cc} & \Delta l_{\text{1}}=H-\rho_{\text{1}}\theta \\ & \Delta l_{\text{2}}=H-\rho_{\text{2}}\theta \\ & \Delta l_{\text{3}}=\rho_{\text{3}}\theta -H \end{array}\right. \end{array} \end{array}$$ (18)

The resistance torques generated by the silicone tube and the elastic washer are expressed, respectively, as:


$$\begin{array}{c} \begin{array}{c} \sum_{i=\text{1}}^{\text{3}}M_{gi}=\text{2}M_{n\text{1}}+\text{2}M_{n\text{2}}+M_{n\text{3}}+\text{2}k_{n}\left(\Delta l_{\text{3}}h_{\text{3}}-\Delta l_{\text{2}}h_{\text{2}}-\Delta l_{\text{1}}h_{\text{1}}\right) \end{array} \end{array}$$ (19)

$$\begin{array}{c} \begin{array}{c} M_{t\text{1}}+M_{t\text{2}}=\frac{\text{2}k_{t}\left(\Delta l_{\text{2}}h_{\text{2}}+\Delta l_{\text{1}}h_{\text{1}}\right)}{N} \end{array} \end{array}$$ (20)

#### Torque analysis of deformation case ③.

In this case, actuators 1 and 2 undergo elongation, whereas actuator 3 undergoes compression. The driving torque generated by the air pressure acting on the upper end cover of the wrist is expressed as:


$$\begin{array}{c} \begin{array}{c} \sum_{i=\text{1}}^{\text{3}}M_{i}=\text{2}p_{\text{1}}S_{\text{1}}h_{\text{1}}+\text{2}p_{\text{2}}S_{\text{2}}h_{\text{2}}-\text{2}p_{\text{3}}S_{\text{3}}h_{\text{3}} \end{array} \end{array}$$ (21)

From the geometric relationship, it follows that:


$$\begin{array}{c} \begin{array}{c} \left\{ \begin{array}{cc} & \rho_{\text{1}}=(Hcot\theta +h_{\text{1}})\theta \\ & \rho_{\text{2}}=(Hcot\theta +h_{\text{2}})\theta \\ & \rho_{\text{3}}=(Hcot\theta -h_{\text{3}})\theta \end{array}\right.\;\left\{ \begin{array}{cc} & h_{\text{1}}=R_{L}sin(\varphi -\pi /\text{2})\\ & h_{\text{2}}=R_{L}sin(\pi -\varphi +\alpha )\\ & h_{\text{3}}=R_{L}sin(\pi -\varphi -\alpha ) \end{array}\right. \end{array} \end{array}$$ (22)

Within this pivoting range, the deformation compatibility relations among the actuators are expressed as:


$$\begin{array}{c} \begin{array}{c} \left\{ \begin{array}{cc} & \Delta l_{\text{1}}=\rho_{\text{1}}\theta -H\\ & \Delta l_{\text{2}}=\rho_{\text{2}}\theta -H\\ & \Delta l_{\text{3}}=H-\rho_{\text{3}}\theta \end{array}\right. \end{array} \end{array}$$ (23)

The resistance torques generated by the silicone tube and the elastic washer are expressed, respectively, as:


$$\begin{array}{c} \begin{array}{c} \sum_{i=\text{1}}^{\text{3}}M_{ni}=\text{2}M_{n\text{1}}+\text{2}M_{n\text{2}}+\text{2}M_{n\text{3}}+\text{2}k_{n}\left(\Delta l_{\text{1}}h_{\text{1}}+\Delta l_{\text{2}}h_{\text{2}}-\Delta l_{\text{3}}h_{\text{3}}\right) \end{array} \end{array}$$ (24)

$$\begin{array}{c} \begin{array}{c} M_{t\text{3}}=\frac{\text{2}k_{t}\Delta l_{\text{3}}h_{\text{3}}}{N} \end{array} \end{array}$$ (25)

Substituting Eqs. (10)–(25) into Eq. (7) yields the joint pivot angle in Region I. Similarly, the pivot angles of the joint in Regions II and III are obtained in the same manner.

### Theoretical analysis of the braking performance of the pneumatic cross-hinge brake

The pneumatic cross-hinge brake comprises two structurally identical braking units arranged orthogonally. This arrangement enables real-time multidirectional braking with adjustable torque.

Fig 5 illustrates the braking principle of the brake in a single direction. When compressed air is supplied to the braking airbag, the airbag expands. Because the sleeve constrains radial expansion, the airbag elongates axially. Once the pressure reaches a threshold, axial elongation of the airbag eliminates the clearance between the friction plate and the braking ball. As the pressure increases further, the friction plate contacts the outer surface of the braking ball, thereby generating a normal force and the friction needed to constrain motion. To simplify the braking-torque model, the following two assumptions are adopted: (1) the friction plate is made of aluminum alloy, and the influence of elastic deformation on the contact area is neglected; and (2) the contact surface between the inner surface of the friction plate and the outer surface of the braking ball is assumed to form a conformal spherical pair of equal diameter.

**Fig 5:**
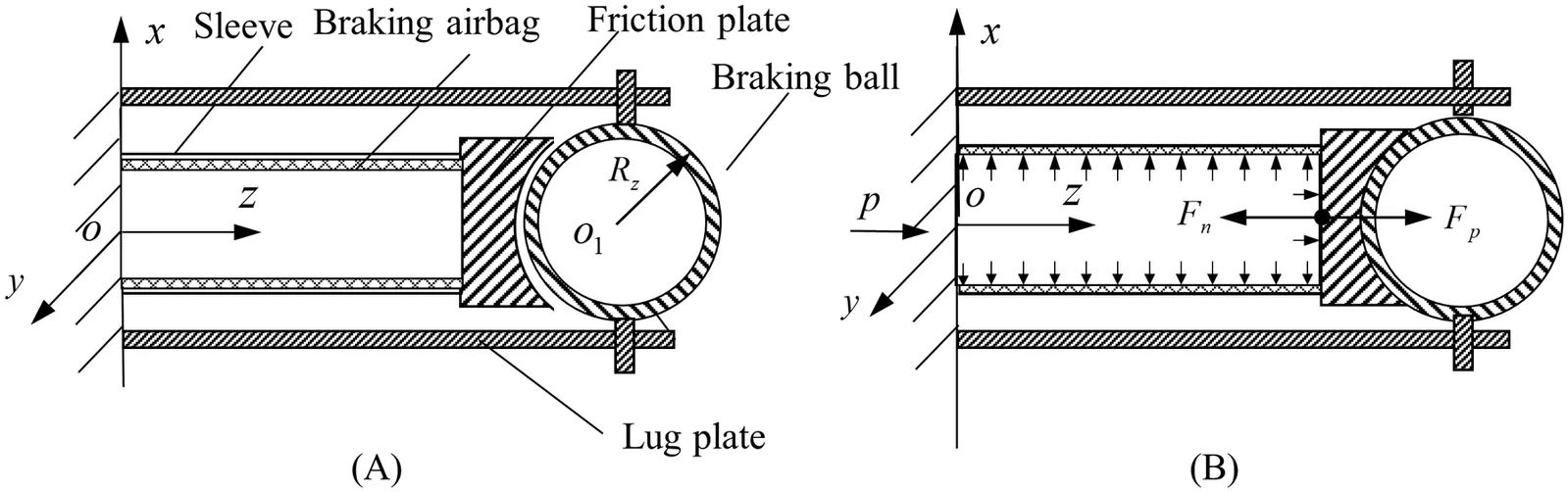
Braking principle of the pneumatic cross-hinge brake. (A) before airbag pressurization and (B) after airbag pressurization.

Force equilibrium in Fig 5B yields the following relationship:


$$\begin{array}{c} \begin{array}{c} F_{d}=F_{p}-F_{n} \end{array} \end{array}$$ (26)

where $F_{d}$ denotes the outward force generated by the braking airbag, and $F_{n}=\frac{E\pi (D_{\text{1}}^{\text{2}}-D_{\text{2}}^{\text{2}})l_{\text{0}}}{\text{4}(l_{\text{0}}+\Delta l{)}^{\text{2}}}\Delta l$ represents the resistance force caused by the axial deformation of the silicone tube itself [36].

To account for the clearances between the braking airbag and the sleeve, as well as between the friction plate and the braking ball, a deformation coordination coefficient is introduced to correct the calculated output force.


$$\begin{array}{c} \begin{array}{c} F_{p}=c_{\text{1}}pS \end{array} \end{array}$$ (27)

where $c_{\text{1}}$ denotes the deformation coordination coefficient of the braking airbag, which depends on the contact conditions at the interfaces among the airbag, sleeve, and friction plate.

Substitution of Eq. (27) into Eq. (26) yields the output force generated by the pressurized braking airbag:


$$\begin{array}{c} \begin{array}{c} F_{d}=\frac{c_{\text{1}}p\pi \left(D_{\text{1}}^{\text{2}}\Delta l+D_{\text{2}}^{\text{2}}l_{\text{0}}\right)}{\text{4}\left(l_{\text{0}}+\Delta l\right)}-\frac{\pi E\left(D_{\text{1}}^{\text{2}}-D_{\text{2}}^{\text{2}}\right)l_{\text{0}}\Delta l}{\text{4}(l_{\text{0}}+\Delta l{)}^{\text{2}}} \end{array} \end{array}$$ (28)

After the friction plate contacts the braking ball, the friction force generated at the interface is expressed as follows:


$$\begin{array}{c} \begin{array}{c} F_{f}=\mu_{\text{1}}F_{d} \end{array} \end{array}$$ (29)

where $\mu_{\text{1}}=\text{0}.\text{3}\text{2}$ is the coefficient of friction between the friction plate and the braking ball, which was determined experimentally.

The braking torque generated at the interface is expressed as follows:


$$\begin{array}{c} \begin{array}{c} M_{z}=\mu F_{d}R_{z}=\mu_{\text{1}}\left[\frac{c_{\text{1}}\pi p\left(D_{\text{1}}^{\text{2}}\Delta l+D_{\text{2}}^{\text{2}}l_{\text{0}}\right)}{\text{4}\left(l_{\text{0}}+\Delta l\right)}-\frac{\pi E\left(D_{\text{1}}^{\text{2}}-D_{\text{2}}^{\text{2}}\right)l_{\text{0}}\Delta l}{\text{4}(l_{\text{0}}+\Delta l{)}^{\text{2}}}\right]R_{z} \end{array} \end{array}$$ (30)

where $M_{z}$ is the braking torque resulting from contact between the friction plate and the braking ball, and $R_{z}$ is the radius of the braking ball.

## Experimental evaluation of wrist mechanical performance

The pneumatic actuators and brake are the principal components that determine the mechanical performance of the flexible wrist. Their structural parameters are summarized in Tables 1 and 2. Using the experimental setup shown in Fig 6, actuator elongation and output force under different limiting planes were measured. The brake torque, wrist pivot angle, pivoting direction angle, and dynamic responses under different excitation conditions were also evaluated. During the experiments, the input pressure was regulated by a pneumatic control system and monitored in real time using a pressure sensor to ensure accurate control.

**Table 1:** Structural parameters of the actuator.

Parameter name	Value	Unit
Total length	120	mm
Operating pressure	[0, 0.30]	MPa
Mass	88	g
Effective deformation length	110	mm
Silicone tube initial outer diameter	15	mm
Silicone tube initial inner diameter	12	mm
Silicone tube elastic modulus	1.042	MPa
Constraint ring thickness	2	mm
Elastic washer inner diameter	15	mm
Elastic washer outer diameter	17	mm
Elastic washer thickness	2	mm
Distances from each of the two artificial muscles to the actuator center	15	mm
Upper and lower connecting end cover thickness	7	mm

**Table 2:** Structural parameters of the pneumatic cross-hinge brake.

Parameter name	Value	Unit
Silicone tube total length	32	mm
Silicone tube effective deformation length	25	mm
Silicone tube initial inner diameter	20	mm
Silicone tube initial outer diameter	24	mm
Sleeve inner diameter	25	mm
Sleeve outer diameter	30	mm
Sleeve height	35	mm
Clearance between the friction plate and the brake ball	0.5	mm
Brake ball radius	20	mm
Distance from braking-ball center to upper and lower end covers	60	mm
Distance from the four lug plates to the braking-ball center	54	mm
Upper and lower end cover thickness	3	mm
Pneumatic cross-hinge brake mass	460	g
Operating pressure	[0, 0.40]	MPa

**Fig 6:**
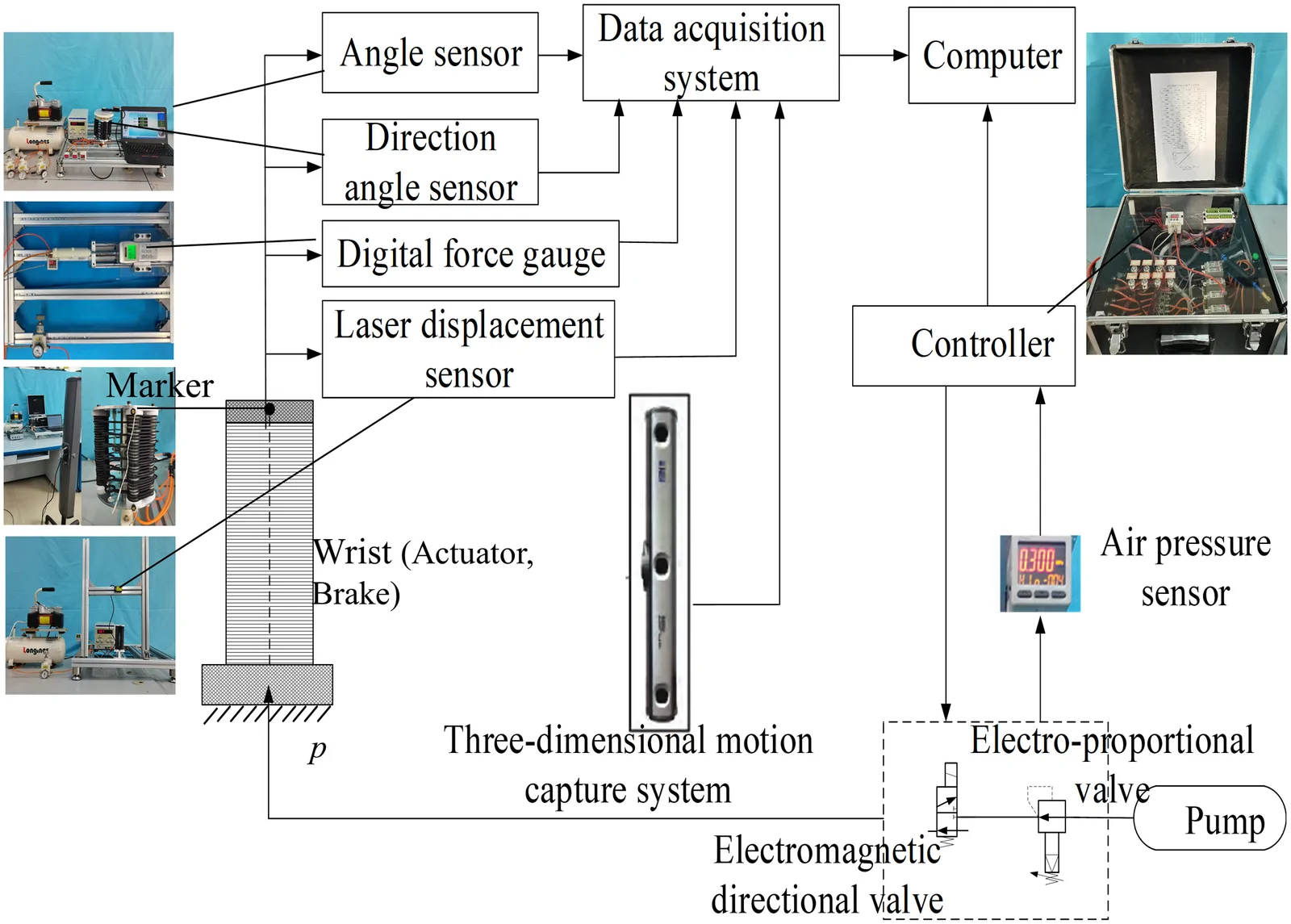
Integrated mechanical experimental system for the wrist.

### Elongation and axial driving force of the actuator

Using the experimental setup shown in Fig 6, actuator elongation was measured as a function of air pressure (Fig 7). The elongation of the actuator increases monotonically with increasing air pressure, showing a clear nonlinear response. At 0.16 MPa, the maximum elongation is 98.2 mm, corresponding to an elongation ratio of 89.3%. The hyperelasticity of the silicone tube produces inherent hysteresis. Consequently, actuator elongation is slightly greater during depressurization than during pressurization.

**Fig 7:**
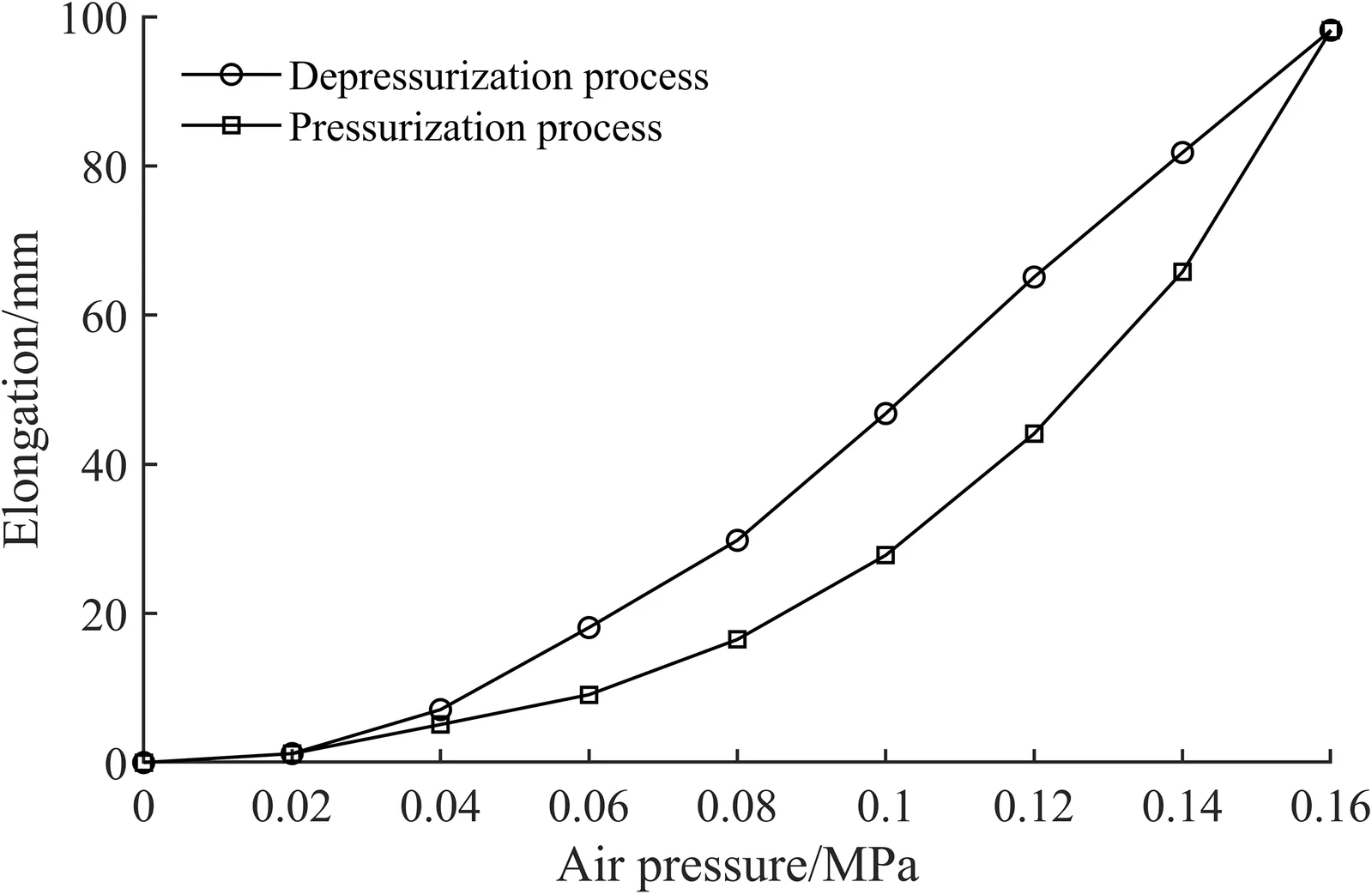
Variation of elongation with air pressure.

Along the axial motion direction of the actuator, five limiting planes were placed at equal intervals to represent different actuator elongations and were denoted Δl=0mm,5.2mm,28mm,82mm,160mm. For each limiting plane, the following procedure was used: (1) the internal pressure of the artificial muscle was adjusted with a precision regulator until the actuator contacted the limiting plane; (2) the sliding stage was moved until the force-sensor probe formed surface-to-surface contact with the actuator’s upper end cover; (3) the air pressure was further increased in increments of 0.05 MPa; and (4) the corresponding axial driving force was recorded.

Fig 8 plots the actuator’s axial driving force as a function of air pressure at the different limiting planes. At all limiting planes, the axial driving force increases approximately linearly with air pressure, although its magnitude varies among the limiting planes. The driving force is inversely related to actuator elongation: a smaller elongation produces a greater axial force. Accordingly, the maximum axial driving force occurs at Δl=0, reaching 69.9 N at 0.30 MPa.

**Fig 8:**
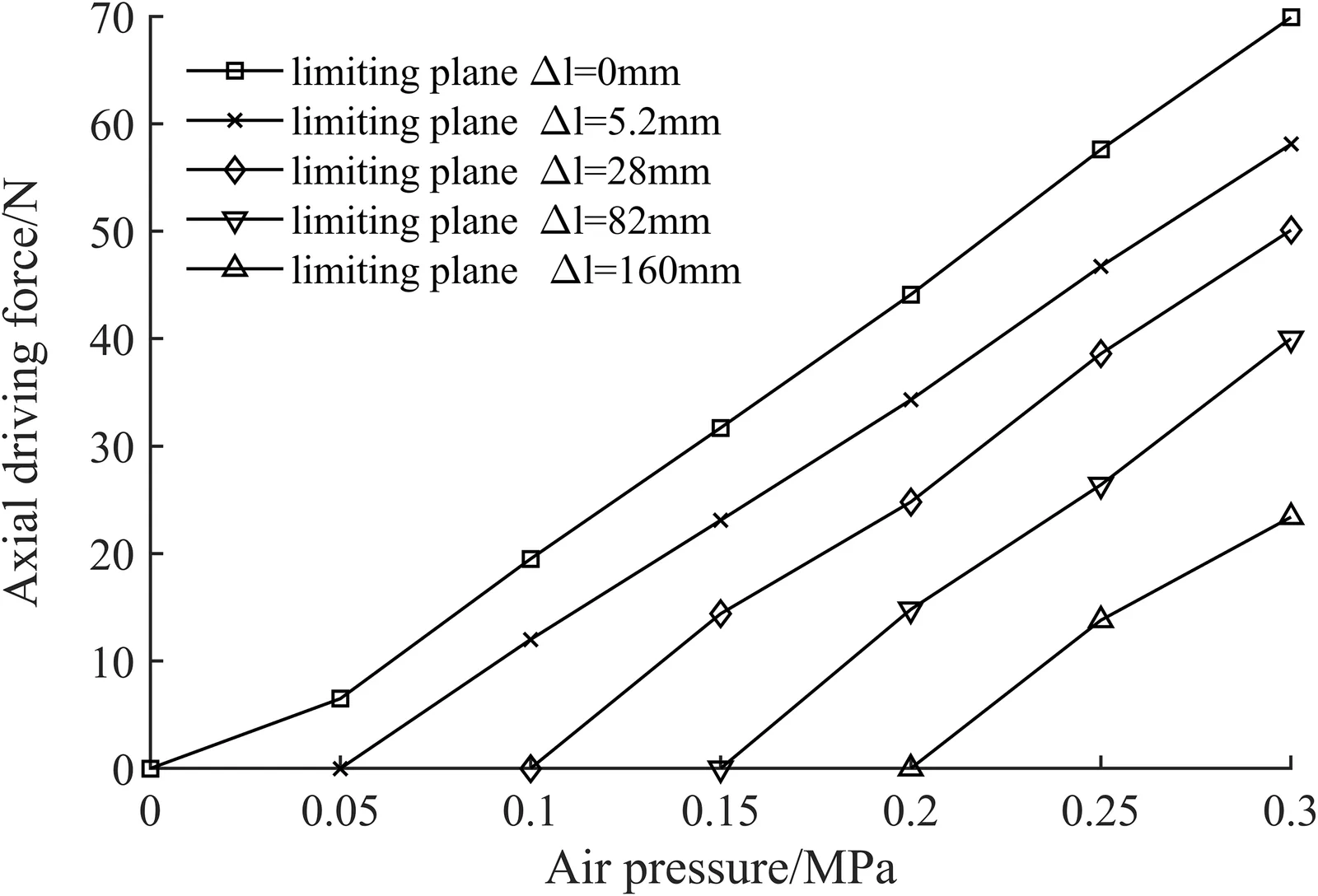
Driving force versus air pressure under different limiting planes.

### Spatial pivoting experiments

To minimize the effects of gravity on the wrist pivot angle and direction, the wrist was mounted vertically on the test platform. A pivoting-direction indicator and an electronic digital angle gauge were mounted at the center of the upper end cover, with the zero line aligned with the positive x-axis. The angle sensor was fixed to the rotating disk of the digital angle gauge. A precision pressure regulator controlled the actuator-chamber pressure and thus the wrist pivot angle and direction. The sensors simultaneously measured the pivoting direction angle and pivot angle.

#### Pivot angle experiments.

In the theoretical model, the compression process of the elastic washers was simplified by neglecting the effects of volumetric compression and variations in shape factor on the axial stiffness. In addition, the integrated braking structure constrains the overall axial elongation of the wrist, causing the deformation of the actuator on the compressed side to deviate from the ideal circular-arc assumption and introducing non-ideal geometric deviations. Since the actuator is primarily composed of soft materials with low bending stiffness, the constraint-induced non-uniform deformation becomes increasingly pronounced with increasing driving pressure. Consequently, the discrepancy between the actual bending profile and the circular-arc assumption gradually increases, thereby reducing the prediction accuracy of the theoretical model for the wrist pivot angle.

Fig 9 shows the comparison curves between theoretical and experimental wrist pivot angles. Two conclusions can be drawn from Fig 9: (1) both the uncorrected theoretical predictions and experimental measurements increase with increasing driving pressure. However, the uncorrected theoretical values consistently overestimate the experimental results, and the absolute deviation between them increases monotonically with pressure, reaching a maximum value of 16.1° at 0.30 MPa; and (2) When the driving pressure is below 0.20 MPa, the measured pivot angle increases nonlinearly with pressure. When the pressure exceeds 0.20 MPa, the pivot angle continues to increase approximately linearly with pressure, but at a lower rate than at lower pressures. These pressure–angle characteristics describe the mechanical response of the wrist but do not, by themselves, validate the circular-arc deformation assumption. The increasing discrepancy between the uncorrected theoretical predictions and experimental measurements at higher pressures may be associated with enhanced mechanical constraints and non-uniform deformation of the soft actuator.

**Fig 9:**
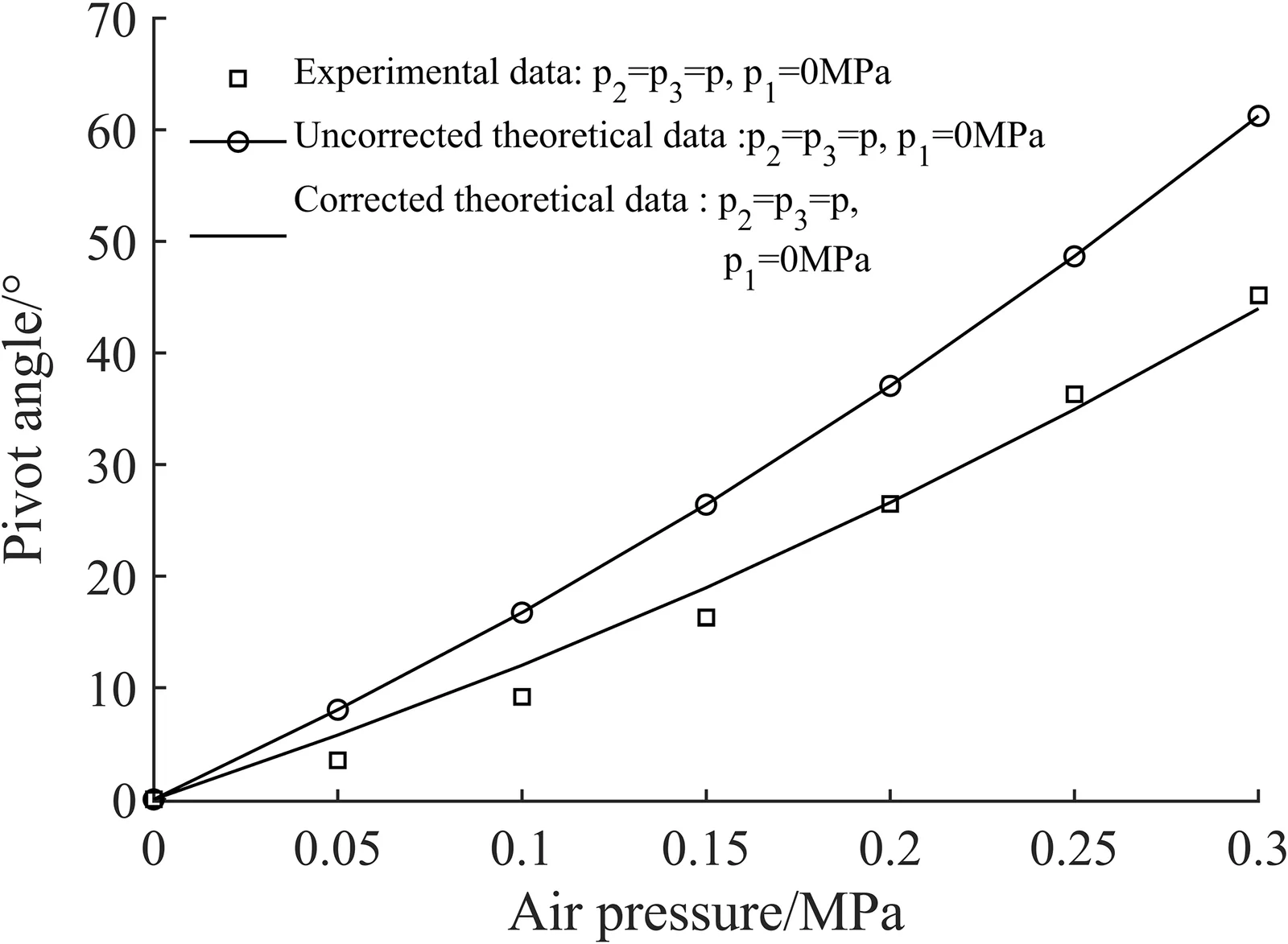
Comparison of theoretical and experimental pivot angles.

To compensate for the above deviation, a proportional correction coefficient of $c_{\text{0}}=\text{0}.\text{7}\text{1}\text{8}$ was determined using the least-squares method based on the experimentally measured pivot-angle data. The same experimental dataset was used to determine *c*_0_ and evaluate the corrected model; therefore, the observed agreement reflects calibration performance rather than independent validation. As shown in Fig 9, introducing this correction coefficient significantly reduces the prediction error. Even at the relatively high driving pressure of 0.30 MPa, the prediction deviation is reduced from 16.1° to 1.2°. Across the entire experimental pressure range, the corrected theoretical results exhibit good agreement with the experimental trend, demonstrating good calibration performance of the corrected model within 0–0.30 MPa. The wrist pivot angle increases continuously with air pressure and reaches 45.2° when $p_{\text{1}}=\text{0}\;\text{MPa},\;p_{\text{2}}=p_{\text{3}}=\text{0}.\text{3}\text{0}\;\text{MPa}$. However, this correction coefficient depends on the structural parameters, constraint conditions, and material properties of the current prototype and therefore should not be regarded as a universal model parameter.

Although the empirical correction effectively reduces the discrepancy between theoretical predictions and experimental measurements, it cannot fundamentally eliminate the underlying physical sources of error. At higher driving pressures, intensified mechanical constraints and non-uniform deformation of the soft material may further weaken the validity of the circular-arc assumption, resulting in reduced model prediction accuracy.

Varying the pressures of the three actuators changes the wrist pivoting direction, as shown in Fig 10. Fig 10 shows that when $p_{\text{1}}$ is kept constant, the pivot angle increases nonlinearly as $p_{\text{2}}$ and $p_{\text{3}}$ increase. The pivot angle ranges from 0° to 45.2°. The maximum pivot angle occurs when $p_{\text{1}}=\text{0}\;\text{MPa},\;p_{\text{2}}=p_{\text{3}}=\text{0}.\text{3}\;\text{MPa}$, whereas the minimum pivot angle is 0° and occurs when $p_{\text{1}}=p_{\text{2}}=p_{\text{3}}=\text{0}\;\text{MPa}$. Under this condition, the resultant torque acting on the wrist is zero.

**Fig 10:**
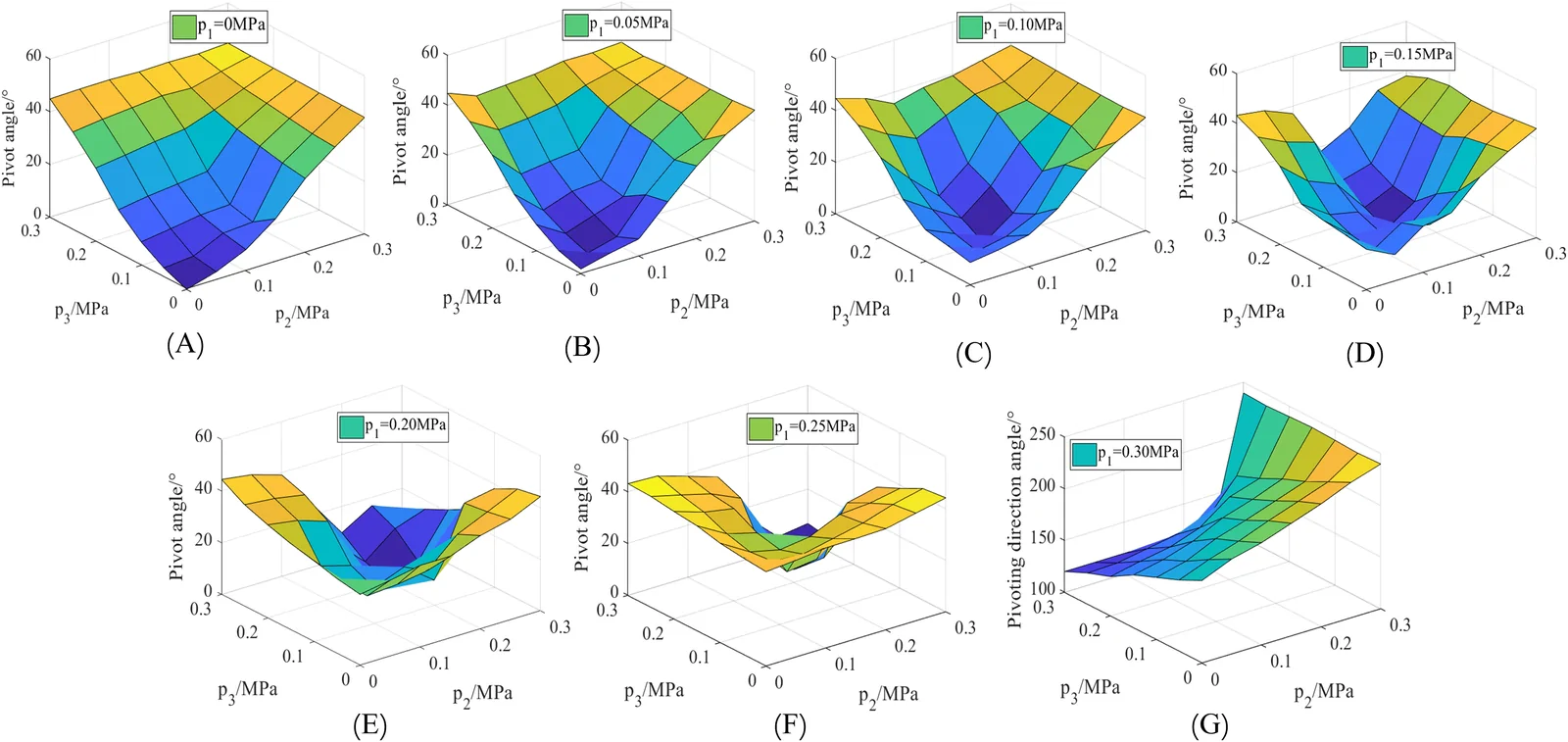
Response surface of wrist pivot angle with respect to air pressure. (A) $p_{\text{1}}=\text{0}\;\text{MPa}$. (B) $p_{\text{1}}=\text{0}.\text{0}\text{5}\;\text{MPa}$. (C) $p_{\text{1}}=\text{0}.\text{1}\text{0}\;\text{MPa}$. (D) $p_{\text{1}}=\text{0}.\text{1}\text{5}\;\text{MPa}$. (E) $p_{\text{1}}=\text{0}.\text{2}\text{0}\;\text{MPa}$. (F) $p_{\text{1}}=\text{0}.\text{2}\text{5}\;\text{MPa}$. (G) $p_{\text{1}}=\text{0}.\text{3}\text{0}\;\text{MPa}$.

#### Pivoting direction angle experiments.

The initial wrist pivoting direction was defined as the positive x -axis, with counterclockwise rotation taken as positive. Substituting the structural parameters of the wrist into Eq. (6) yields the pressure-dependent pivoting direction angle plotted in Fig 11.

**Fig 11:**
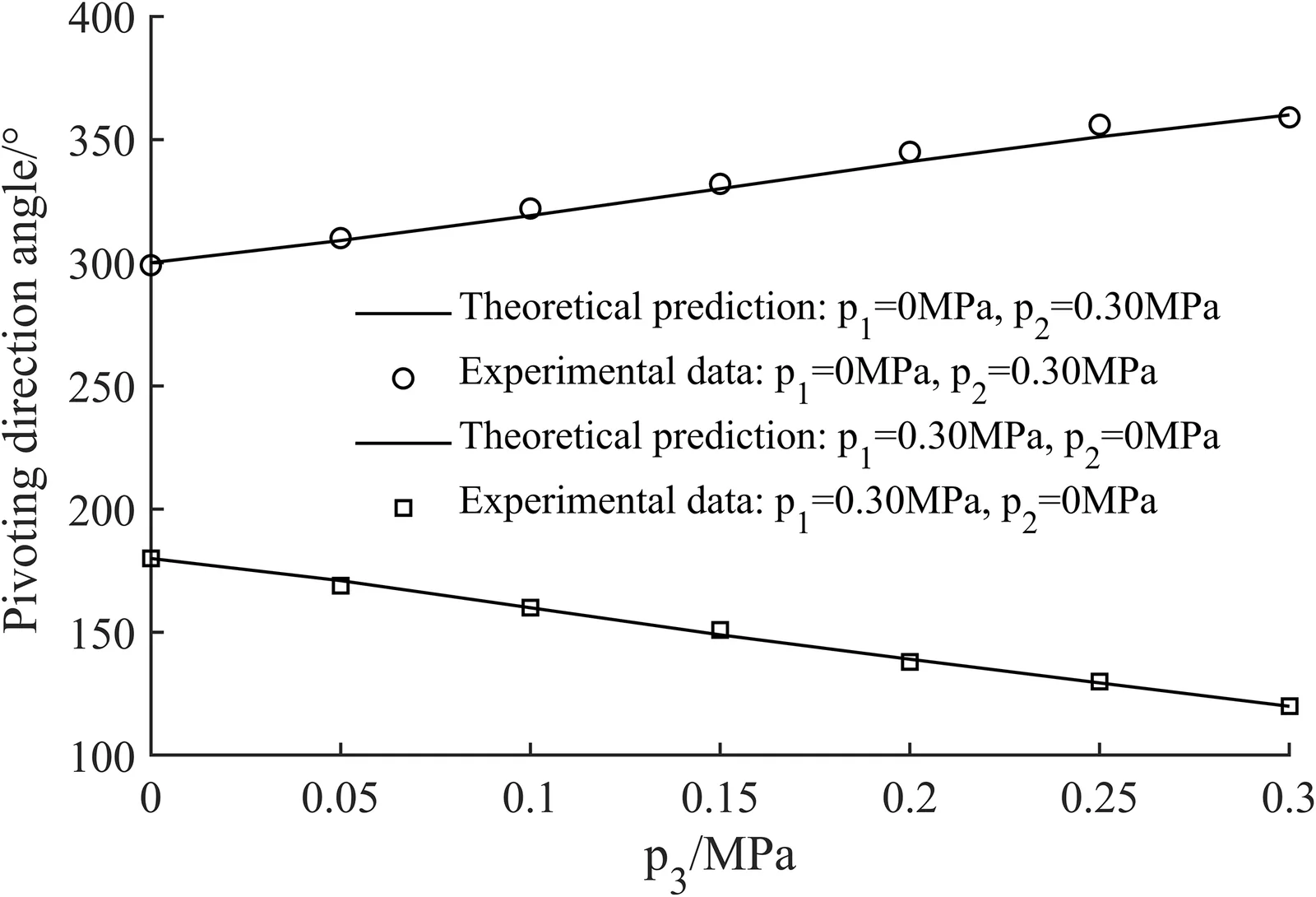
Comparison between theoretical and experimental pivoting direction angle curves.

As shown in Fig 11, the theoretical predictions closely match the experimental data. The model therefore accurately captures the pressure-dependent spatial pivoting direction angle. When the pressures in $p_{\text{1}}$ and $p_{\text{2}}$ are kept constant, the pivoting direction angle varies as $p_{\text{3}}$ increases.

When $p_{\text{1}}$ is kept constant, the wrist can pivot in any direction in the xoy plane from 0° to 360° by adjusting $p_{\text{2}}$ and $p_{\text{3}}$, as shown in Fig 12. In Fig 12A–12G, six boundary lines appear during wrist motion over the range of 0° to 360°, namely 0° (360°), 60°, 120°, 180°, 240°, and 300°. In Fig 12A, when $p_{\text{1}}=\text{0}\;\text{MPa},\;p_{\text{2}}<p_{\text{3}}$, the wrist pivoting direction angle changes gradually within 0° to 60°; when $p_{\text{1}}=\text{0}\;\text{MPa},\;p_{\text{2}}>p_{\text{3}}$, it varies within 300° to 360°. The two trends are symmetric about the x-axis. As $p_{\text{1}}$ increases from 0 MPa to 0.30 MPa, the response surface of the wrist pivoting direction angle becomes smoother.

**Fig 12:**
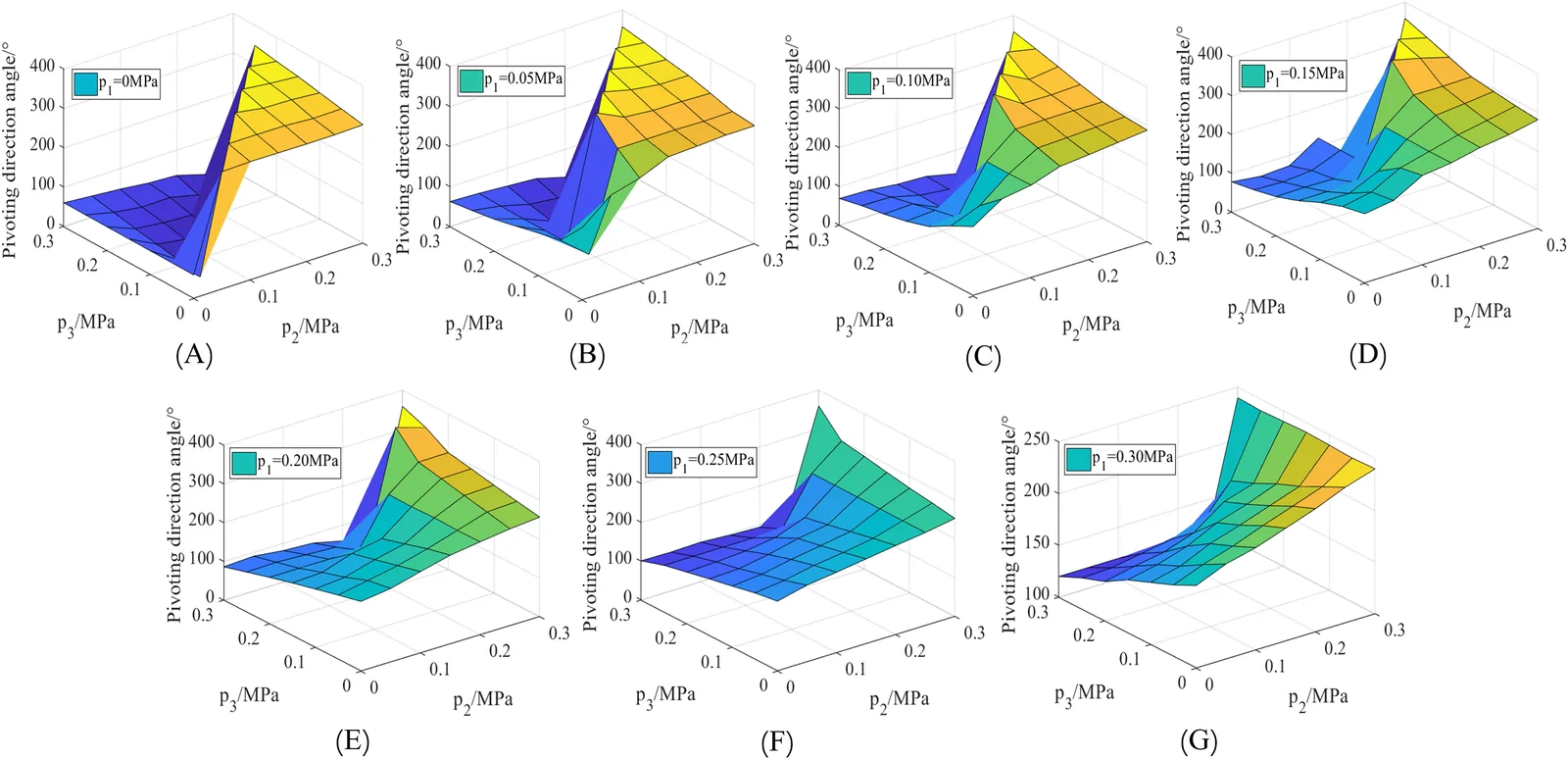
Response surface of wrist pivoting direction angle with respect to air pressure. (A) $p_{\text{1}}=\text{0}\;\text{MPa}$. (B) $p_{\text{1}}=\text{0}.\text{0}\text{5}\;\text{MPa}$. (C) $p_{\text{1}}=\text{0}.\text{1}\text{0}\;\text{MPa}$. (D) $p_{\text{1}}=\text{0}.\text{1}\text{5}\;\text{MPa}$. (E) $p_{\text{1}}=\text{0}.\text{2}\text{0}\;\text{MPa}$. (F) $p_{\text{1}}=\text{0}.\text{2}\text{5}\;\text{MPa}$. (G) $p_{\text{1}}=\text{0}.\text{3}\;\text{MPa}$.

### Experiment on the braking performance of the cross-hinge brake

#### Experiment on the output force of the braking airbag.

The braking airbag was mounted in a 3D-printed fixture whose sleeve dimensions matched those of the brake. During the experiment, the movable stage was adjusted until the probe of the force gauge contacted the upper surface of the braking airbag. The air pressure was then increased incrementally, and the corresponding axial output force was measured with a digital force gauge. The brake parameters in Table 2 were substituted into Eq. (28), and the model predictions were compared with the experimental data. The comparison yielded a deformation coordination coefficient of 1.16 ($c_{\text{1}}$= 1.16), as shown in Fig 13.

**Fig 13:**
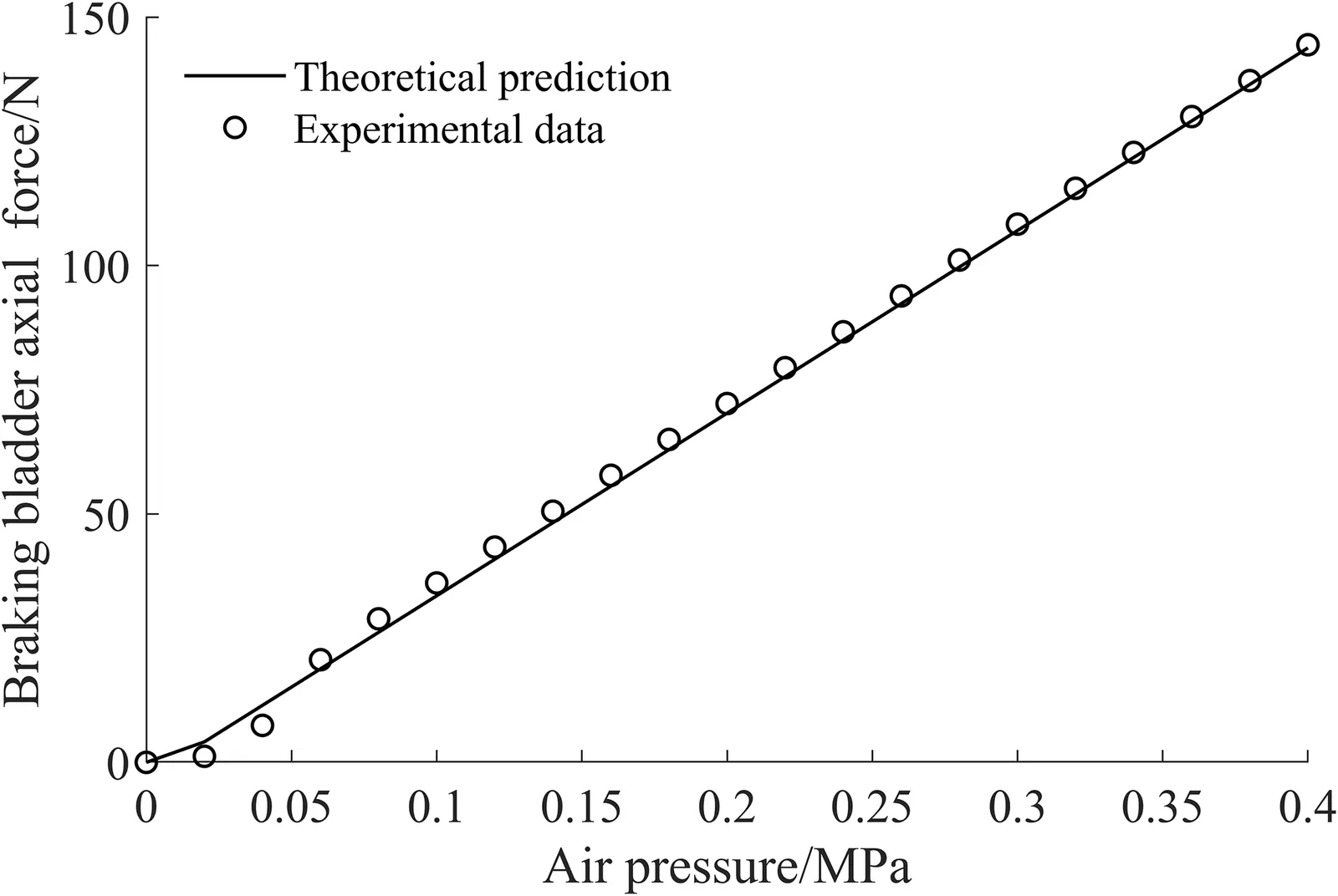
Comparison of theoretical and experimental axial output forces of the braking airbag.

Fig 13 shows that (1) the theoretical predictions agree closely with the experimental data, indicating that the model captures the pressure-dependent axial output force of the braking airbag, and (2) the axial output force increases approximately linearly with air pressure and reaches 144.4 N at 0.40 MPa. Below 0.04 MPa, the measured output force remains small because the supplied air is consumed primarily in expanding the airbag. Once the airbag closes the assembly clearance, the output force rises more rapidly.

#### Braking torque experiments.

Fig 14 illustrates the braking-torque measurement method. To eliminate gravitational effects, the brake was mounted with its axis vertical. The two ends of the cable were connected to the upper end cover of the brake and the center of the digital force gauge, respectively. A pulley kept the upper end cover parallel to the loading direction of the force gauge throughout the test. The experimental setup is shown in Fig 15. During the experiment, the air pressure inside the brake chamber was adjusted with a precision pressure regulator to vary the friction between the friction plate and the braking ball. The force-gauge load was increased continuously, while an angle sensor measured the corresponding pivot angle. When the pivot angle changed abruptly, the corresponding force reading was recorded as the maximum sustainable load at that pressure.

**Fig 14:**
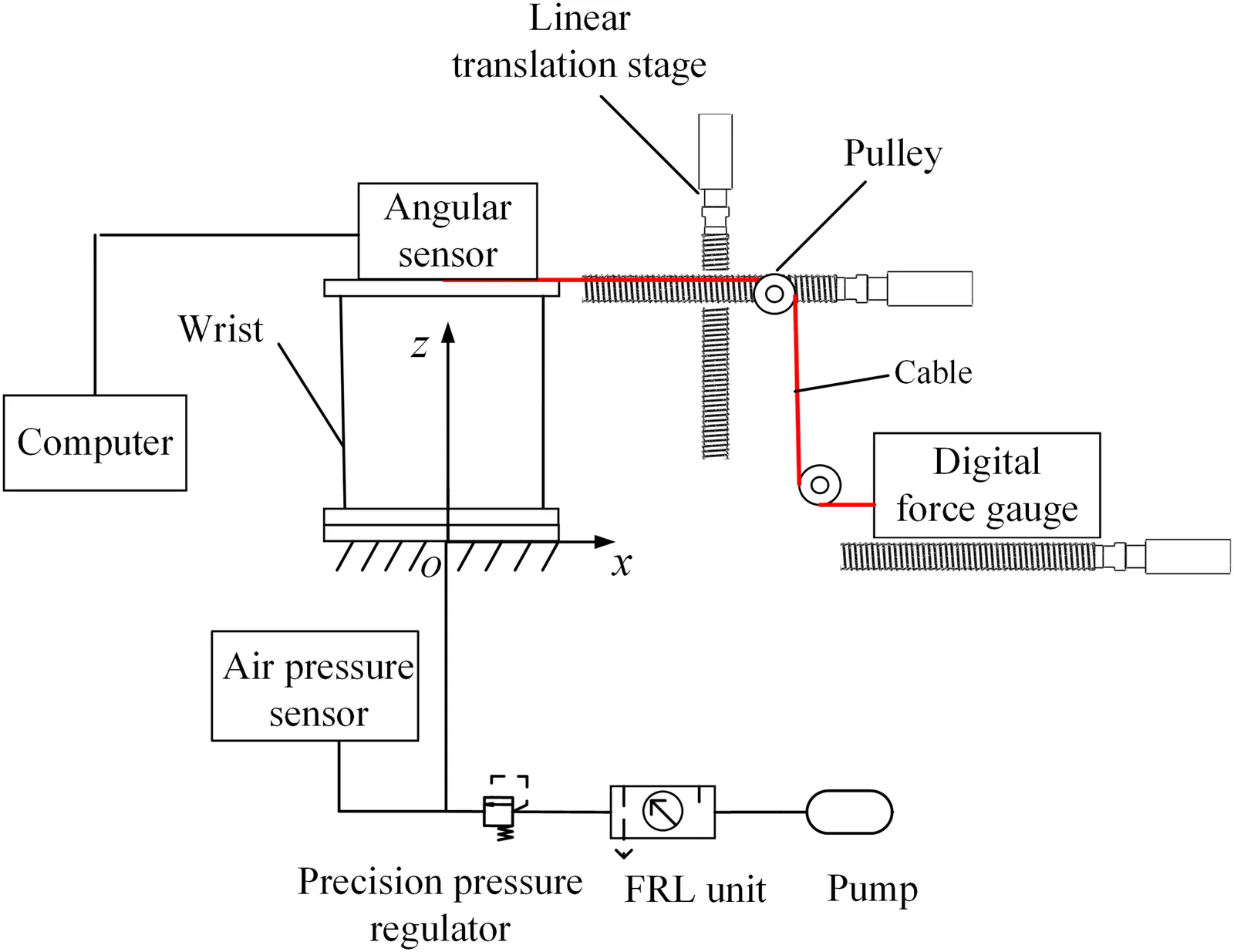
Schematic of the braking-torque experiment.

**Fig 15:**
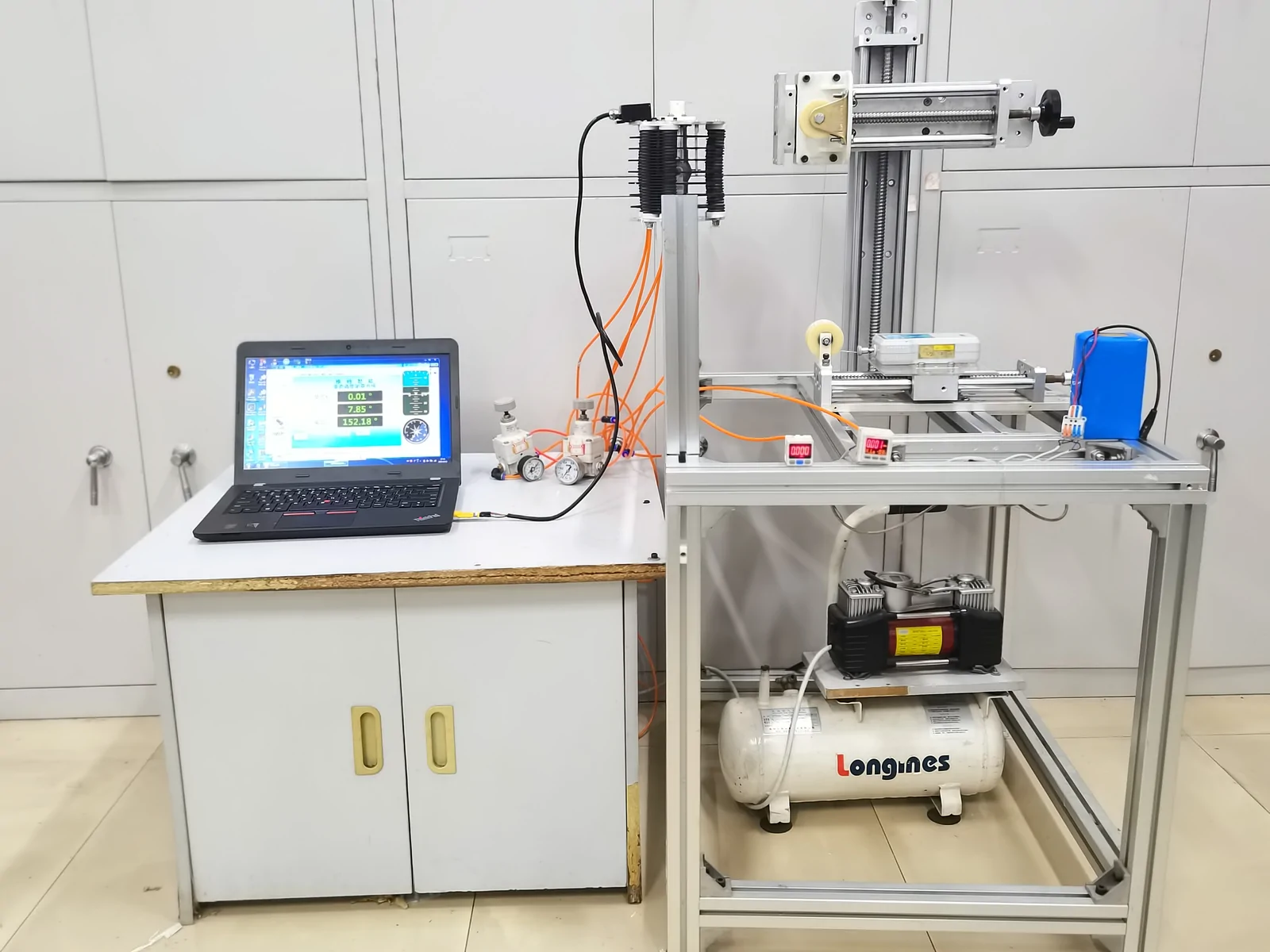
Experimental setup for braking torque.

The unidirectional braking torque was calculated by substituting the structural parameters of the brake into Eq. (30) and compared with the experimental data (Fig 16). Fig 16 shows that (1) under the tested conditions, the braking torque provided by the brake alone increases approximately linearly with the air pressure in the braking airbag, reaching a maximum measured value of 0.842 N·m at 0.35 MPa; and (2) the close agreement between the theoretical and experimental results supports the proposed braking-torque model.

**Fig 16:**
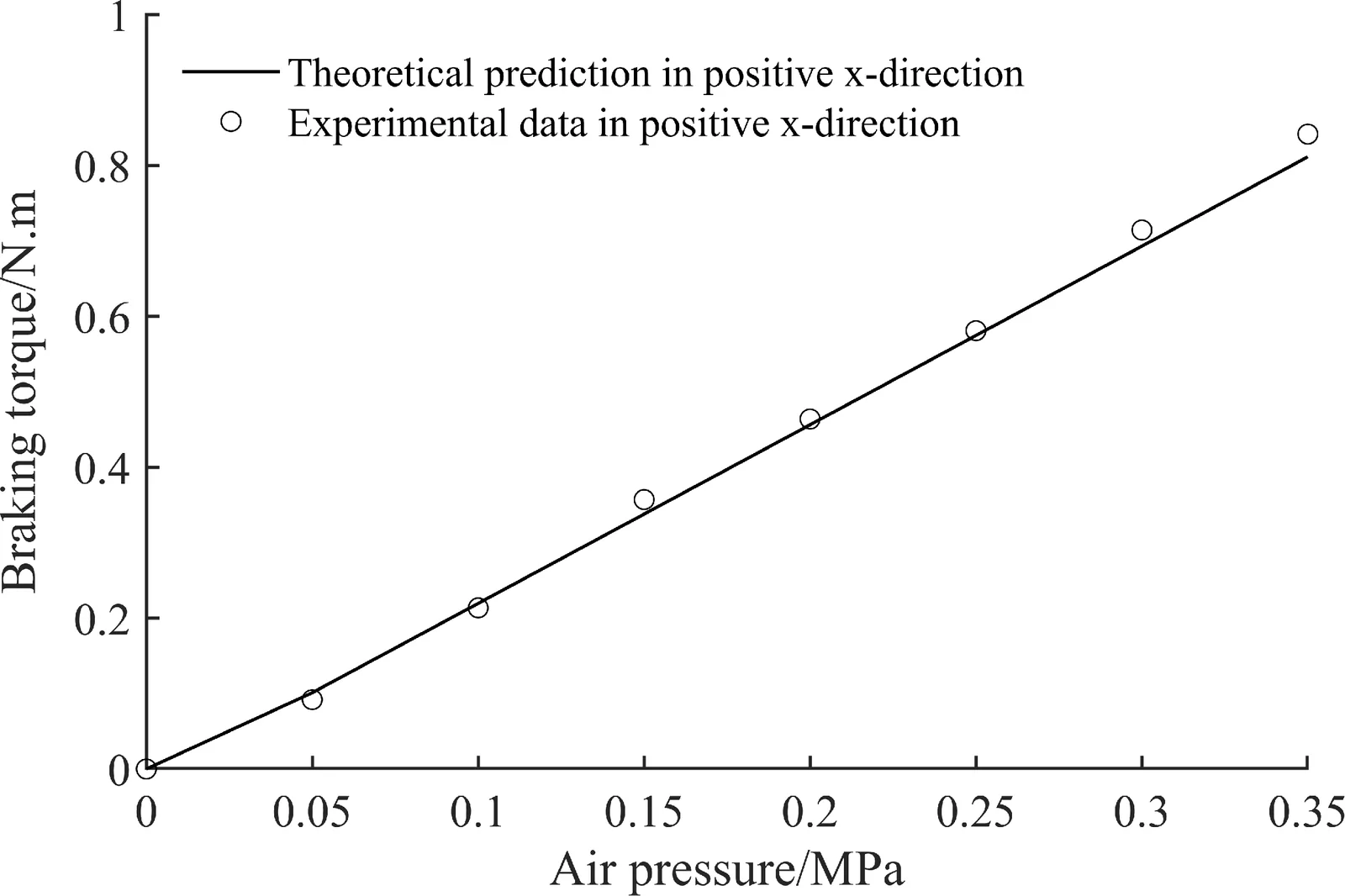
Comparison of theoretical and experimental braking torque.

Fig 17 compares the braking torques measured in two orthogonal directions (the positive x -axis is perpendicular to the axis of actuator No. 1). The small difference between the two directions indicates that the loading direction has little effect on the braking capacity.

**Fig 17:**
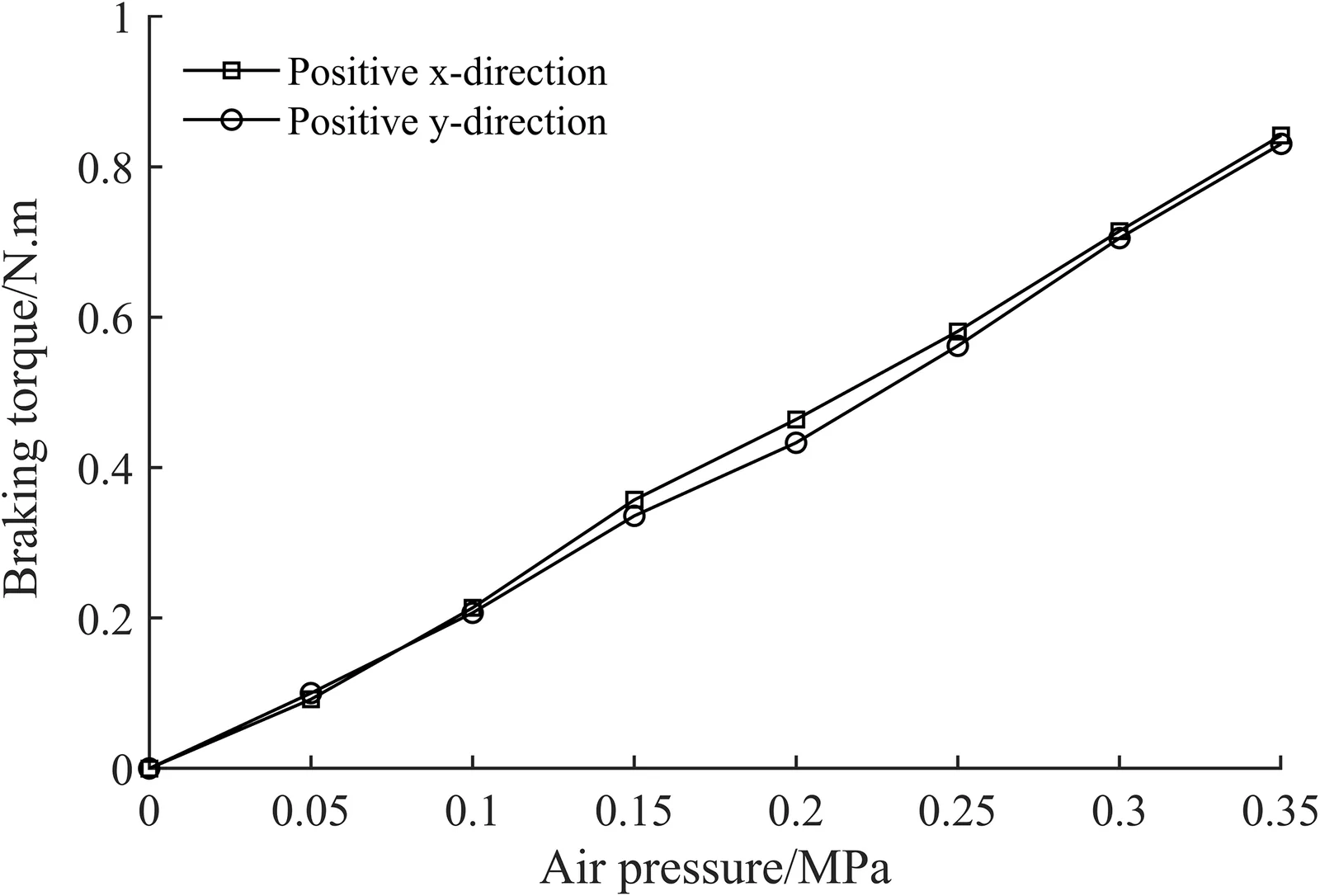
Comparison of braking torque in orthogonal directions.

#### Wrist mechanical performance under constant pivot-angle and friction-torque conditions.

The theoretical models of pivot angle and pivoting direction describe wrist deformation under no-load, unbraked conditions. However, the implicit equations are computationally intensive and unsuitable for direct real-time control. To derive simpler control-oriented relationships, experiments under constant-pivot-angle and constant-friction-torque conditions examined the maximum external load resisted by the wrist $M_{zk}$, the pivot angle θ, the friction torque $M_{f}$, and the increment in driving air pressure Δp. Based on these relationships, an impedance-torque model $M_{zk}=f_{\text{1}}(\Delta p,\theta ,\varphi )$ and a pivot-angle model $\theta =f_{\text{2}}(\Delta p,M_{f},\varphi )$ were established.

(1) Effect of braking air pressure on wrist impedance torque at a constant pivot angle

The pivoting direction and pivot angle of the wrist were adjusted in 10° increments by controlling the ventilation combinations of different actuators. The procedure was as follows: ① the pressures of the relevant actuators were adjusted to move the wrist to the prescribed angle; ② the linear stage was positioned so that the loading and pivoting directions coincided; and ③ different braking air pressures were applied, and the maximum external load resisted by the wrist under each braking air pressure was measured using a force gauge.

Polynomial functions were fitted to the data for wrist pivoting along the positive x -axis. For a first-order surface, the fitting error was relatively large, with a maximum error of 0.1 N·m. A second-order surface reduced the maximum error to 0.035 N·m, corresponding to a 65% reduction. Although a third-order surface further reduced the maximum error to 0.019 N·m, it substantially increased equation complexity while providing only a modest gain in accuracy. Therefore, a second-order polynomial was selected for the wrist impedance-torque fit, and the fitted expression is:


$$\begin{array}{c} \begin{array}{c} M_{zk}=f_{\text{1}}(\Delta p,\theta ,\varphi )=a_{\text{0}\text{0}}+a_{\text{1}\text{0}}\Delta p+a_{\text{0}\text{1}}\theta +a_{\text{2}\text{0}}\Delta p^{\text{2}}+a_{\text{1}\text{1}}\Delta p\theta +a_{\text{0}\text{2}}\theta^{\text{2}} \end{array} \end{array}$$ (31)

where $a_{\text{0}\text{0}}=\text{0}.\text{2}\text{5}\text{2},\;a_{\text{1}\text{0}}=\text{1}.\text{4}\text{8}\text{7},a_{\text{0}\text{1}}=\text{0}.\text{0}\text{0}\text{0}\text{4}\text{3},a_{\text{2}\text{0}}=\text{2}.\text{7}\text{7}\text{6},a_{\text{1}\text{1}}=\text{0}.\text{0}\text{0}\text{2}\text{8},a_{\text{0}\text{2}}=\text{7}.\text{6}e-\text{5}$, and the coefficient of determination is 0.9958.

Fig 18 presents the fitted response surface and residual plot of the wrist impedance torque during pivoting in the positive x -axis direction. As shown in Fig 18A, the composite impedance torque obtained under the joint action of the actuators and brake increased from 0.280 to 1.396 N·m with increasing braking and driving pressures, corresponding to a 4.98-fold increase in the wrist’s resistance to external loads. As shown in Fig 18B, the residuals were uniformly distributed around zero, with the maximum error remaining within 0.0393 N·m. These results indicate that the empirical impedance-torque equation is sufficiently accurate for subsequent analysis and control.

**Fig 18:**
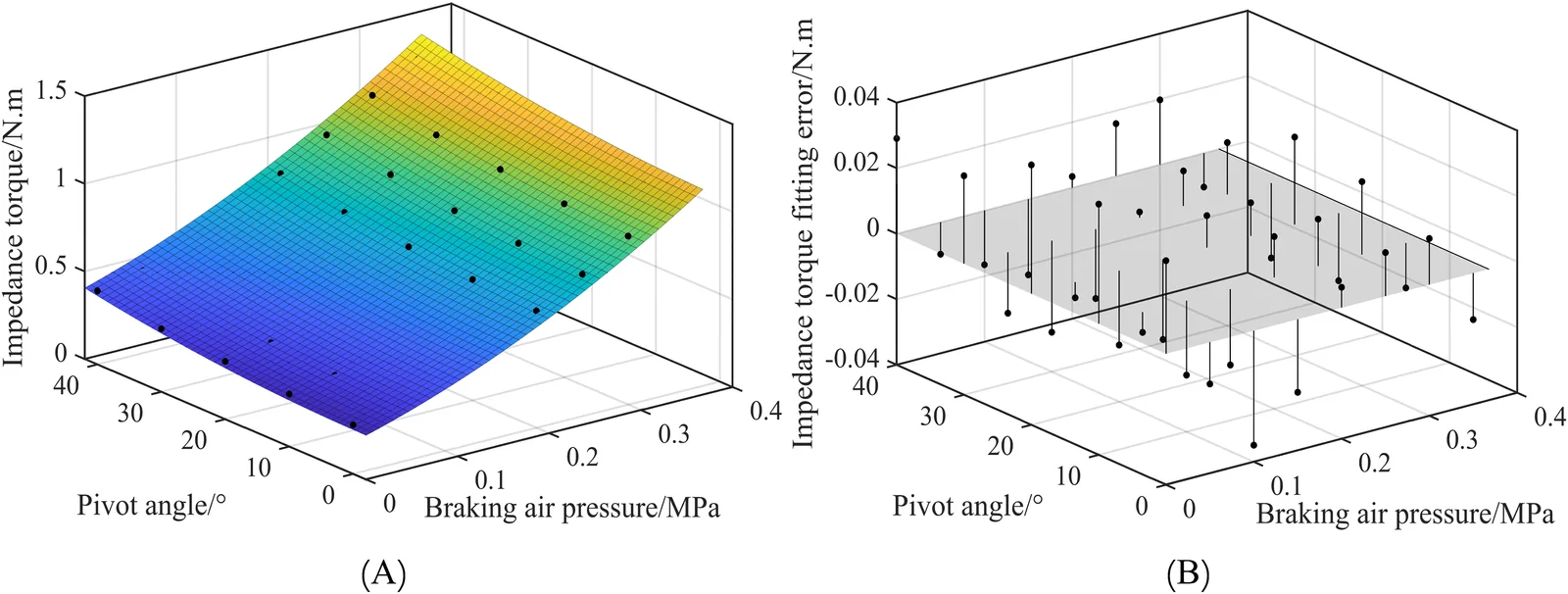
Fitting of wrist impedance torque. (A) Fitting surface. (B) Residual plot.

(2) Effect of actuator pressure on wrist pivot angle under constant friction torque

At a given friction torque, the wrist continues to deform as the actuator pressure increases. Using the experimental setup shown in Fig 15, the braking-airbag pressure was first adjusted in 0.05-MPa increments to prescribe the wrist friction torque. The actuator pressure was then adjusted to deflect the wrist toward the positive x -axis, and an angle sensor recorded the resulting pivot angle. The pivot-angle data at different friction torques were likewise fitted with a second-order polynomial:


$$\begin{array}{c} \begin{array}{c} \theta =f_{\text{1}}\left(\Delta p,M_{f},\varphi \right)=a_{\text{0}\text{0}}+a_{\text{1}\text{0}}\Delta p+a_{\text{0}\text{1}}M_{f}+a_{\text{2}\text{0}}\Delta p^{\text{2}}+a_{\text{1}\text{1}}\Delta pM_{f}+a_{\text{0}\text{2}}{M_{f}}^{\text{2}} \end{array} \end{array}$$ (32)

where $a_{\text{0}\text{0}}=-\text{0}.\text{0}\text{2}\text{6},\;a_{\text{1}\text{0}}=\text{6}\text{6}.\text{9},a_{\text{0}\text{1}}=-\text{5}.\text{5}\text{9}\text{3},a_{\text{2}\text{0}}=\text{3}\text{0}\text{0}.\text{1},a_{\text{1}\text{1}}=-\text{2}\text{6}\text{8}.\text{7},a_{\text{0}\text{2}}=\text{3}\text{1}.\text{2}\text{5}$, and the coefficient of determination is 0.9965.

Fig 19 presents the fitted response surface and residual plot of the wrist pivot angle. Fig 19A shows that the pivot angle decreases nonlinearly as the braking air pressure increases. In Fig 19B, the residuals are distributed uniformly within a narrow range around zero, with a maximum error of 1.8556°. The fitted pivot-angle equation is therefore suitable for subsequent analysis.

**Fig 19:**
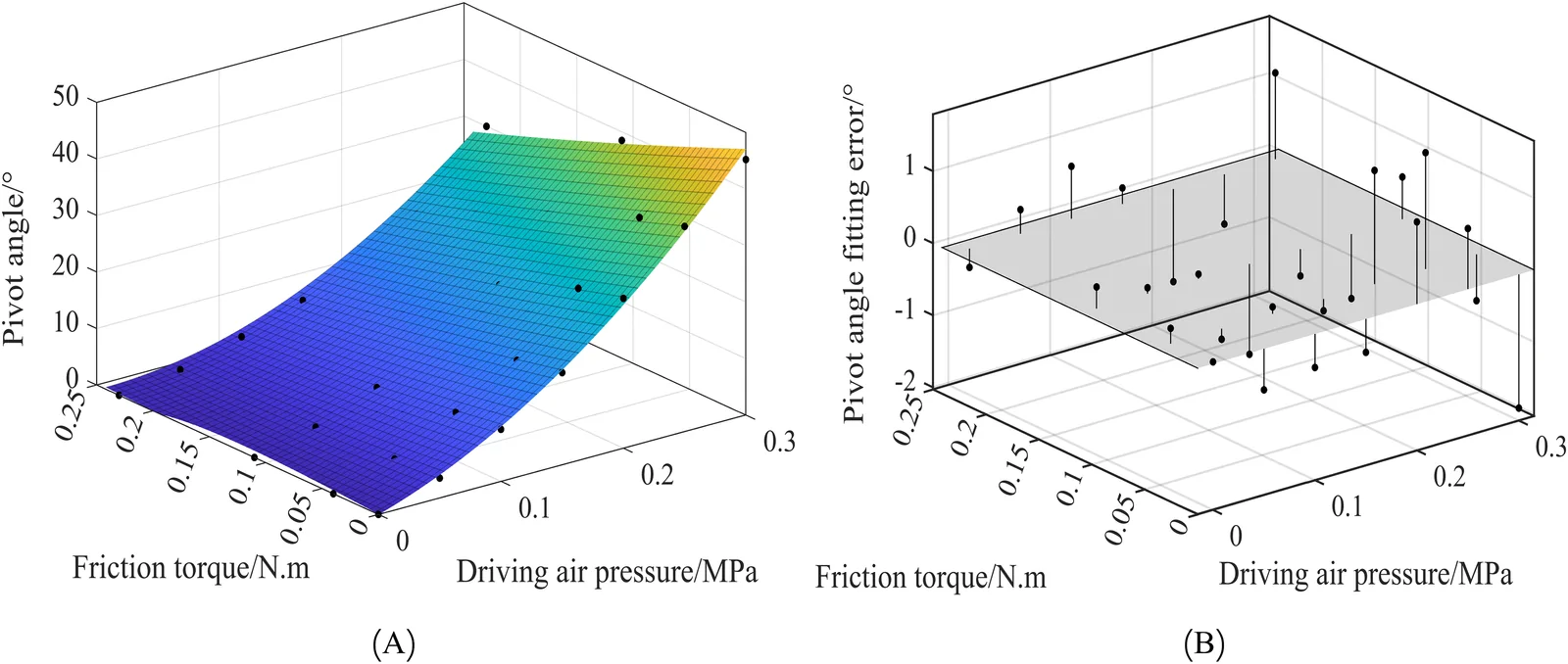
Fitting of wrist pivot angle. (A) Fitting surface. (B) Residual plot.

The same procedure may be used to derive fitted equations for the impedance torque and pivot angle of the wrist in other pivoting directions, supporting accurate real-time control in future studies.

### Experimental evaluation of wrist dynamics

The wrist consists of three actuators connected in parallel with a brake. The resulting system exhibits time-varying parameters, strong nonlinearity, and substantial coupling. These characteristics complicate accurate theoretical modeling. Dynamic experiments under different excitation signals were therefore conducted using the system shown in Fig 6, with brake activation as the single experimental variable. The excitation signals were generated by a programmable logic controller (PLC), and a three-dimensional motion-capture system tracked wrist-mounted markers to obtain time-resolved kinematic data. The detailed experimental conditions are summarized in Table 3.

**Table 3:** Dynamic experimental conditions.

Parameter name	Value	Unit
Pulse duration	0.5, 1.0, 1.5	s
Operating mode	Actuators 2 and 3 pressurized; actuator 1 unpressurized	
Driving air pressure	0.10, 0.20, 0.30	MPa
Braking air pressure	0, 0.05, 0.10, 0.15	MPa
Sampling frequency	500	Sps (points·s ⁻ ¹)

#### Time-domain response experiments.

(1) Step-response analysis

Fig 20 shows the time-domain response curves of the wrist under step excitation at different braking air pressures when the driving air pressure is 0.30 MPa. Increasing the braking-air pressure raises the damping, slows the wrist response, and reduces the steady-state pivot angle.

**Fig 20:**
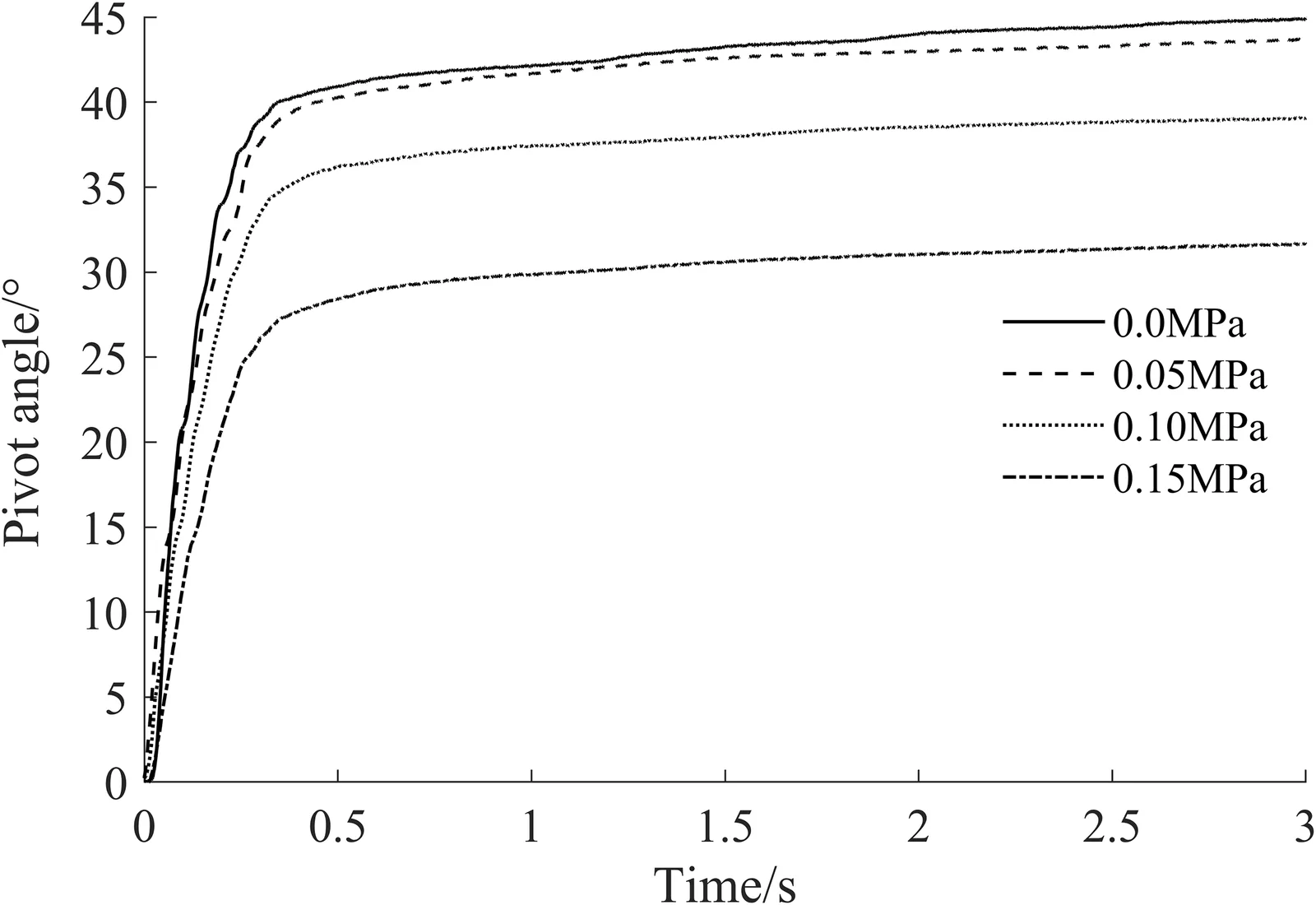
Time-domain step response of the wrist during braking.

(2) Pulse-response analysis

Fig 21 shows the time-domain pulse responses of the wrist without braking. Fig 21A presents the responses under different pulse widths at a driving air pressure of 0.30 MPa. For comparison, Fig 21B shows the responses under different driving air pressures at a pulse width of 1.5 s.

**Fig 21:**
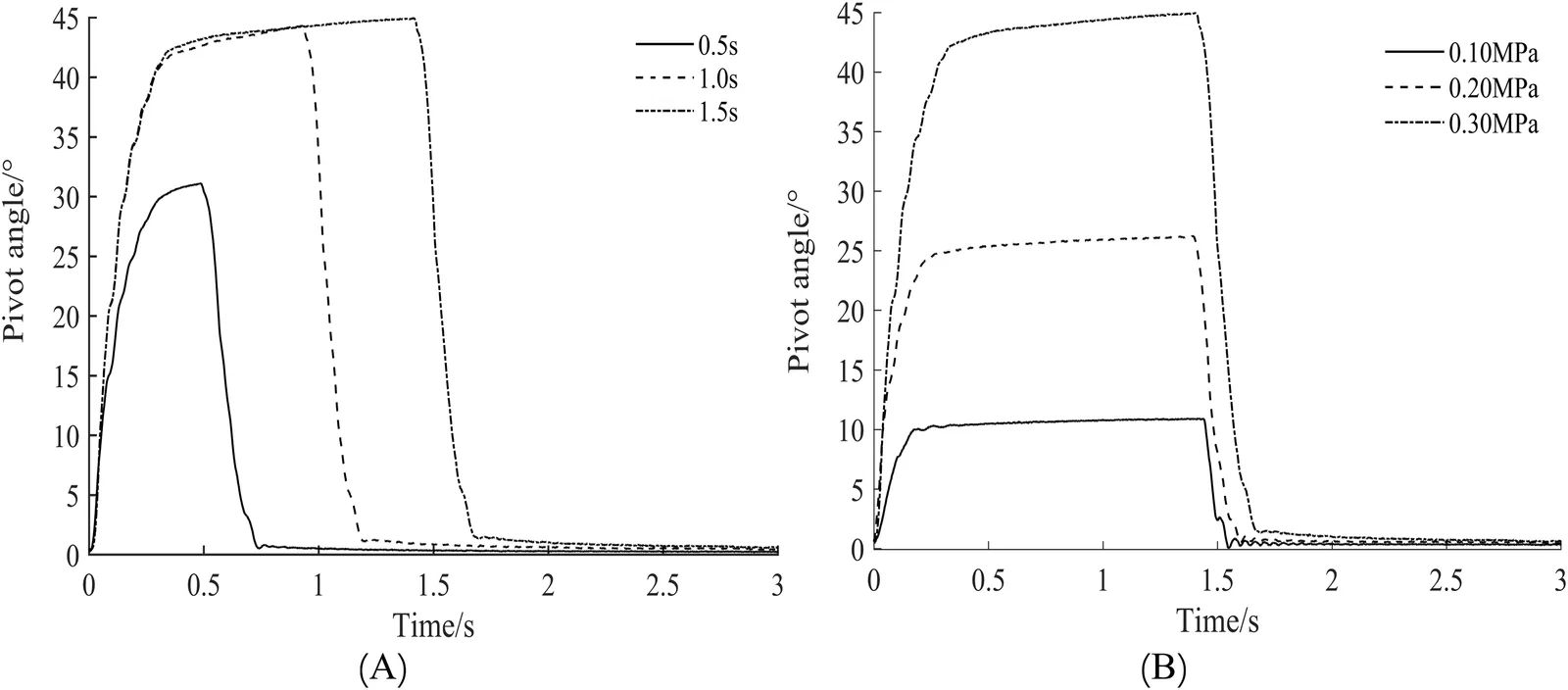
Pulse time-domain responses without braking. (A) Different pulse widths at a constant driving air pressure. (B) Different driving air pressures at a constant pulse width.

As shown in Fig 21A, when the pulse width is shorter than 1.0 s, the pivot angle of the wrist increases continuously with time and does not reach its steady-state value. When the pulse width is 1.5 s, the pivot angle rises more slowly and nearly reaches the steady-state position. Fig 21B shows that the steady-state pivot angle of the wrist increases with increasing driving air pressure. During depressurization, the pneumatic stiffness drops rapidly, reducing the overall wrist stiffness and causing slight oscillation near the initial position.

The time-domain response data shown in Fig 21B were measured under no-payload, non-braked conditions using a pulse-width excitation of 1.5 s at different driving pressures. The data were processed using discrete differentiation to calculate the instantaneous angular velocity at each sampling point. The average forward-pivoting response speed was then determined over the interval from the onset of pressurization to the attainment of the target pressure. The results show that, at target driving pressures of 0.30, 0.20, and 0.10 MPa, the corresponding average angular velocities during forward pivoting were 92.4°/s, 68.8°/s, and 50.2°/s, respectively. It should be noted that these speeds reflect the response speed of the proposed wrist under the specified experimental conditions and should not be interpreted as its general speed advantage over the existing system.

Fig 22 shows the pulse time-domain responses at a pulse width of 1.5 s under different braking conditions. Specifically, Fig 22A shows the responses at different braking air pressures with the driving air pressure fixed at 0.30 MPa, whereas Fig 22B presents the responses at different driving air pressures with the braking air pressure fixed at 0.15 MPa.

**Fig 22:**
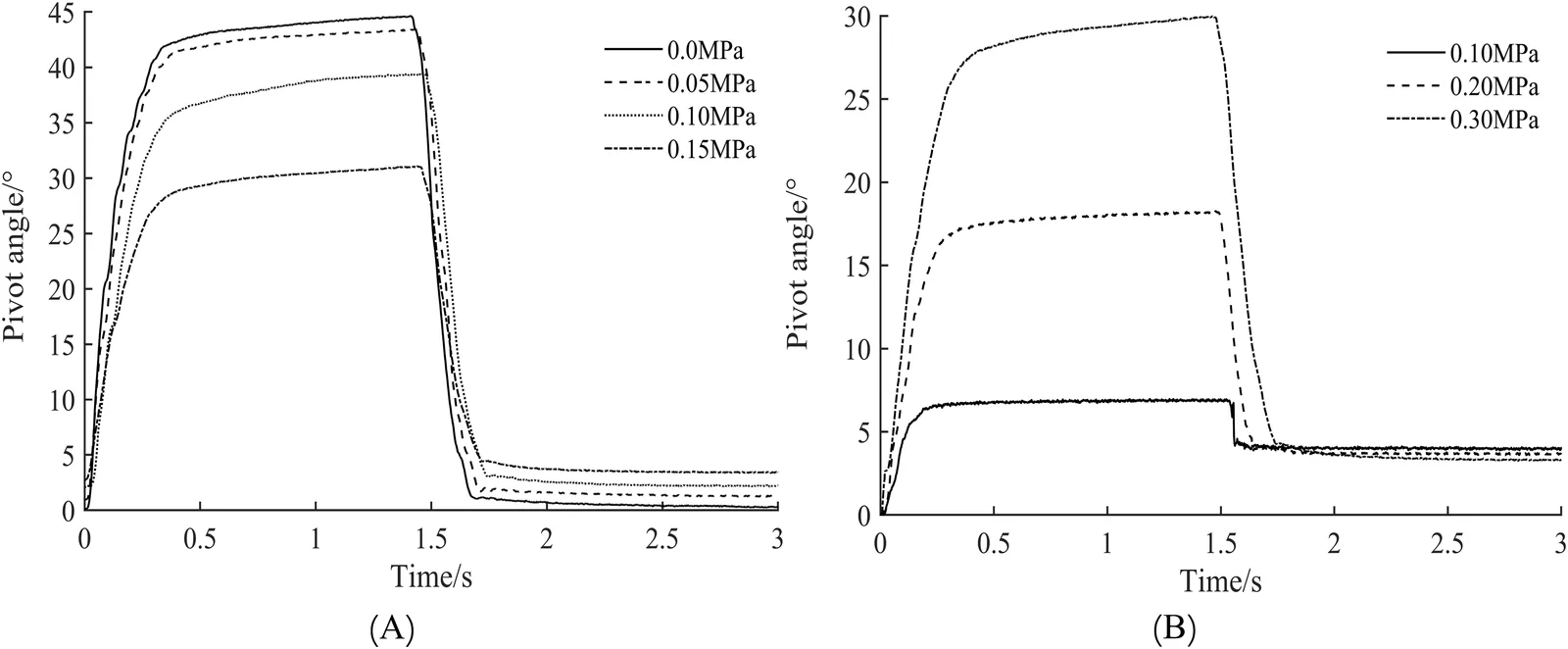
Pulse time-domain responses during braking. (A) Different braking air pressures at a constant driving air pressure. (B) Different driving air pressures at a constant braking air pressure.

Fig 22A shows that the wrist pivot angle decreases as the braking pressure increases. At a braking pressure of 0.15 MPa, the pivot angle is 29.8°, which is 34.11% lower than that without braking. During actuator depressurization, the wrist does not return to its initial position because the brake remains pressurized, leaving a residual angular deviation that increases with braking pressure. This residual deviation occurs because the braking torque, which increases with braking pressure, dissipates part of the energy available for elastic recovery.

Fig 22B shows that at a braking pressure of 0.15 MPa, the reductions in the steady-state wrist pivot angle under different driving pressures are 30.9%, 31.51%, and 34.11%, respectively, relative to the corresponding values without braking. During actuator depressurization, the wrist retains a residual angular deviation from its initial position at steady state, and this deviation gradually decreases as the driving pressure increases. A higher driving pressure produces a larger pivot angle and stores more elastic energy in the silicone tube, which helps the wrist overcome the fixed braking torque during recovery.

#### Limiting frequency tests at different pivot angles without braking.

For a specified pivot angle, the limiting frequency is the highest excitation frequency at which the wrist can move from its initial position to that angle within a single on-off actuation cycle. At higher frequencies, the wrist cannot reach the prescribed angle. The wrist was driven by the pneumatic control system to achieve pivot angles of 15°, 30°, and 45°. Time-resolved wrist motion at different excitation frequencies was recorded using a three-dimensional motion capture system, as shown in Fig 23.

**Fig 23:**
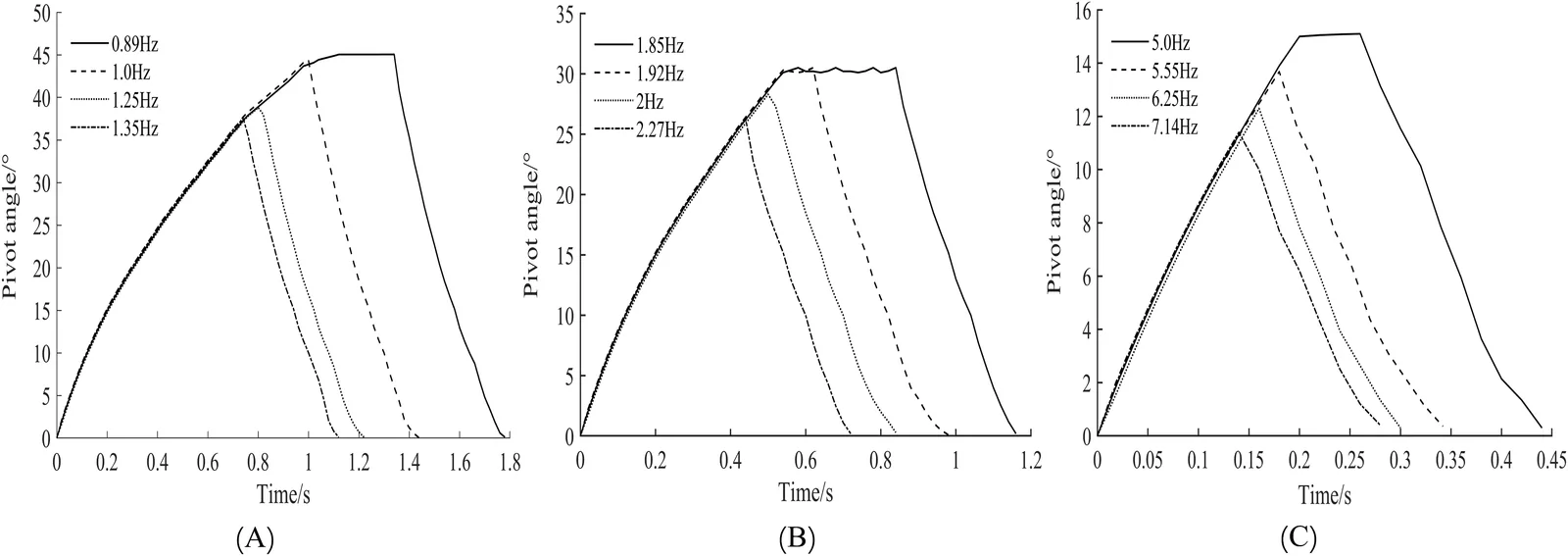
Time response curves of the wrist at different pivot angles: (A) 45°; (B) 30°; and (C) 15°.

Fig 23 shows that a limiting frequency exists for each pivot angle. When the excitation frequency exceeds this limit, the wrist fails to reach the prescribed angle. In Fig 23A, for a pivot angle of 45°, the wrist does not reach 45° under excitation frequencies of 1.0, 1.25, and 1.35 Hz because the available deformation time is insufficient. At 0.89 Hz, the response time is 1.12 s, which allows the wrist to reach and briefly maintain 45°. Thus, the limiting frequency for this angle lies between 0.89 and 1.0 Hz. Similarly, in Fig 23B and 23C, the limiting frequencies lie within 1.92–2.0 Hz and 5.0–5.55 Hz, respectively. More precise limiting frequencies could be determined by narrowing these ranges through bisection.

#### Frequency-domain response analysis.

(1) Frequency-domain response without braking

Fig 24 shows the frequency-domain response of the wrist under step-signal excitation without braking. As shown in Fig 24, the natural frequency lies in the low-frequency range and gradually decreases with increasing driving pressure. This trend mainly reflects the pressure-induced elongation of the silicone tube, which reduces the overall stiffness of the wrist, thereby lowering its natural frequency.

**Fig 24:**
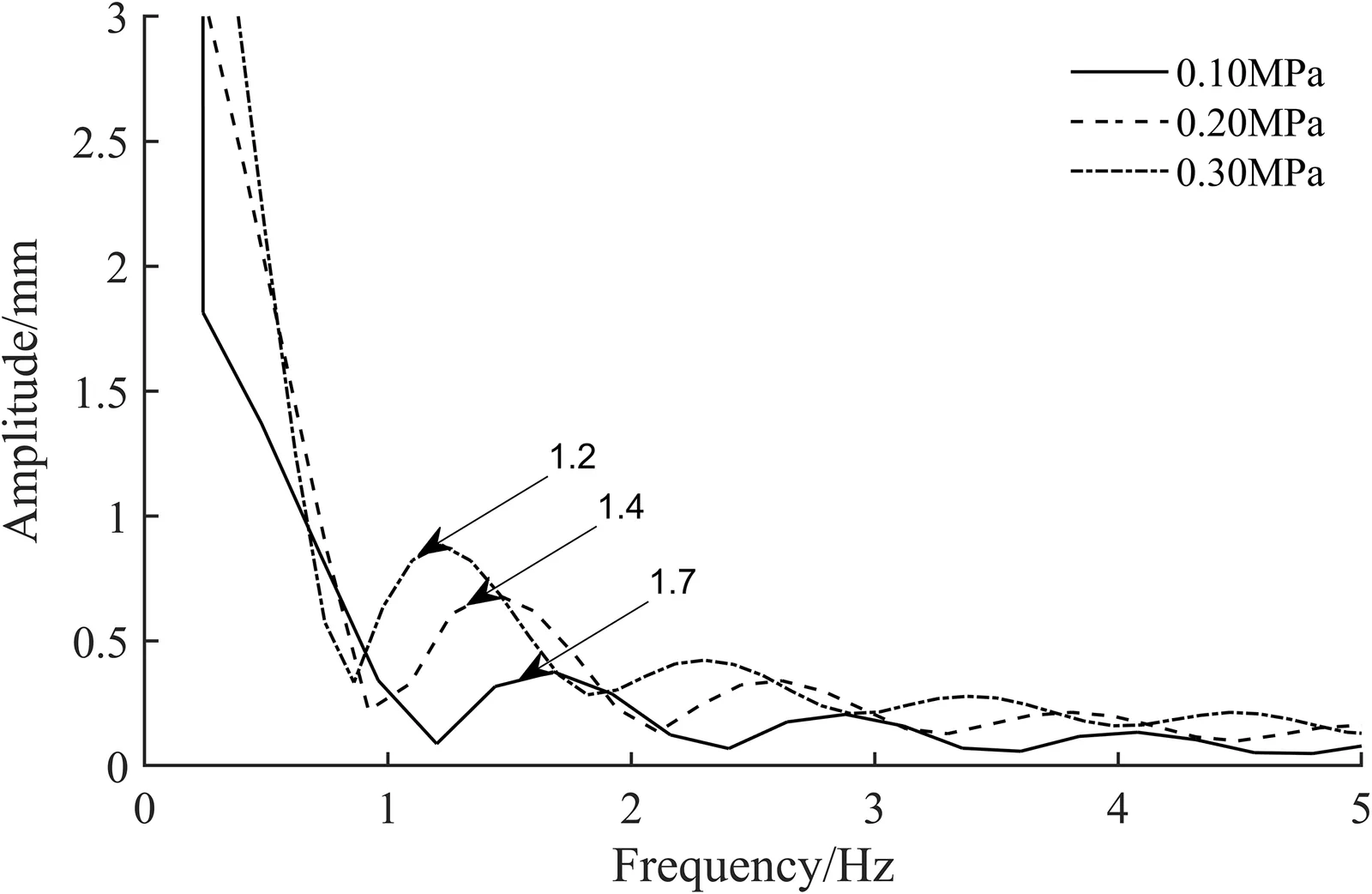
Frequency-domain response of the wrist without braking under step excitation.

A Fourier transform was applied to data recorded during wrist depressurization under pulse excitation. Fig 25 shows the effects of pulse width and driving pressure on the natural frequency.

**Fig 25:**
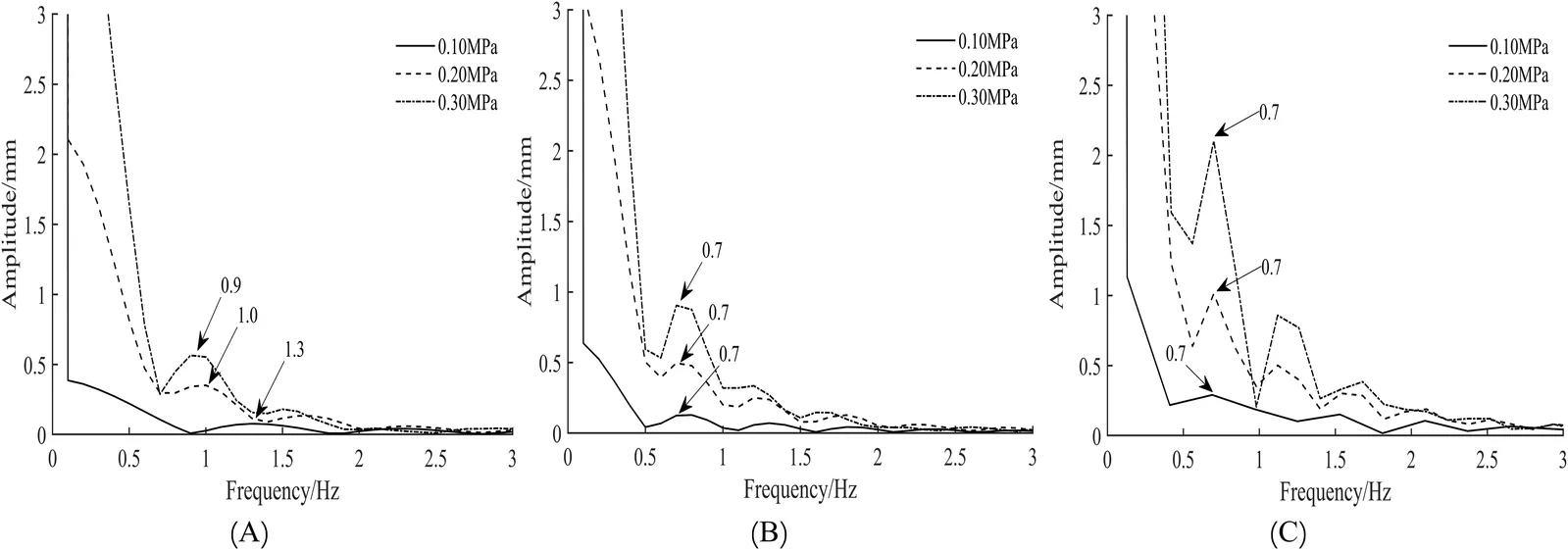
Frequency-domain responses of the wrist without braking under different pulse widths. (A) 0.5 s. (B) 1.0 s. (C) 1.5 s.

As shown in Fig 25, the effect of pulse width on natural frequency exhibits a clear threshold. When the pulse width is 0.5 s, which is below the threshold, the wrist pivot angle does not reach a steady state, and the natural frequency is influenced by both pulse width and driving pressure. In contrast, at pulse widths of 1.0 s and 1.5 s, the pivot angle reaches steady state, and the natural frequency remains constant at 0.7 Hz, indicating that it is independent of pulse width and driving pressure under steady-state conditions. Moreover, the wrist response amplitude increases with driving pressure, as greater silicone-tube deformation at higher pressure produces a larger recovery impact.

(2) Frequency-domain response with braking

Fig 26 illustrates the effect of braking pressure on the amplitude-frequency characteristics of the wrist under step excitation at a driving pressure of 0.30 MPa, whereas Fig 27 presents the corresponding results under pulse excitation with a pulse width of 1.5 s.

**Fig 26:**
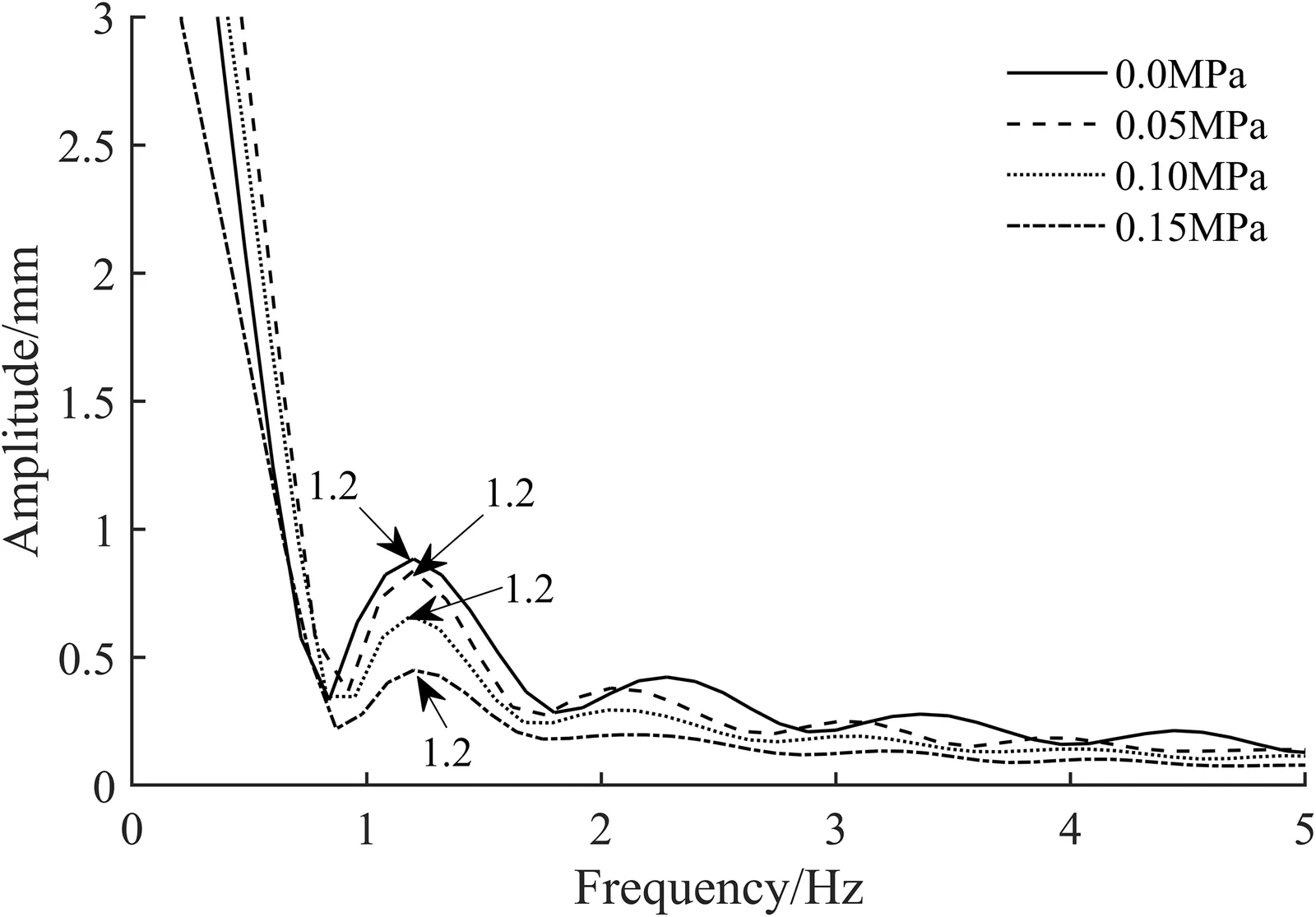
Step frequency-domain responses under different braking pressures.

**Fig 27:**
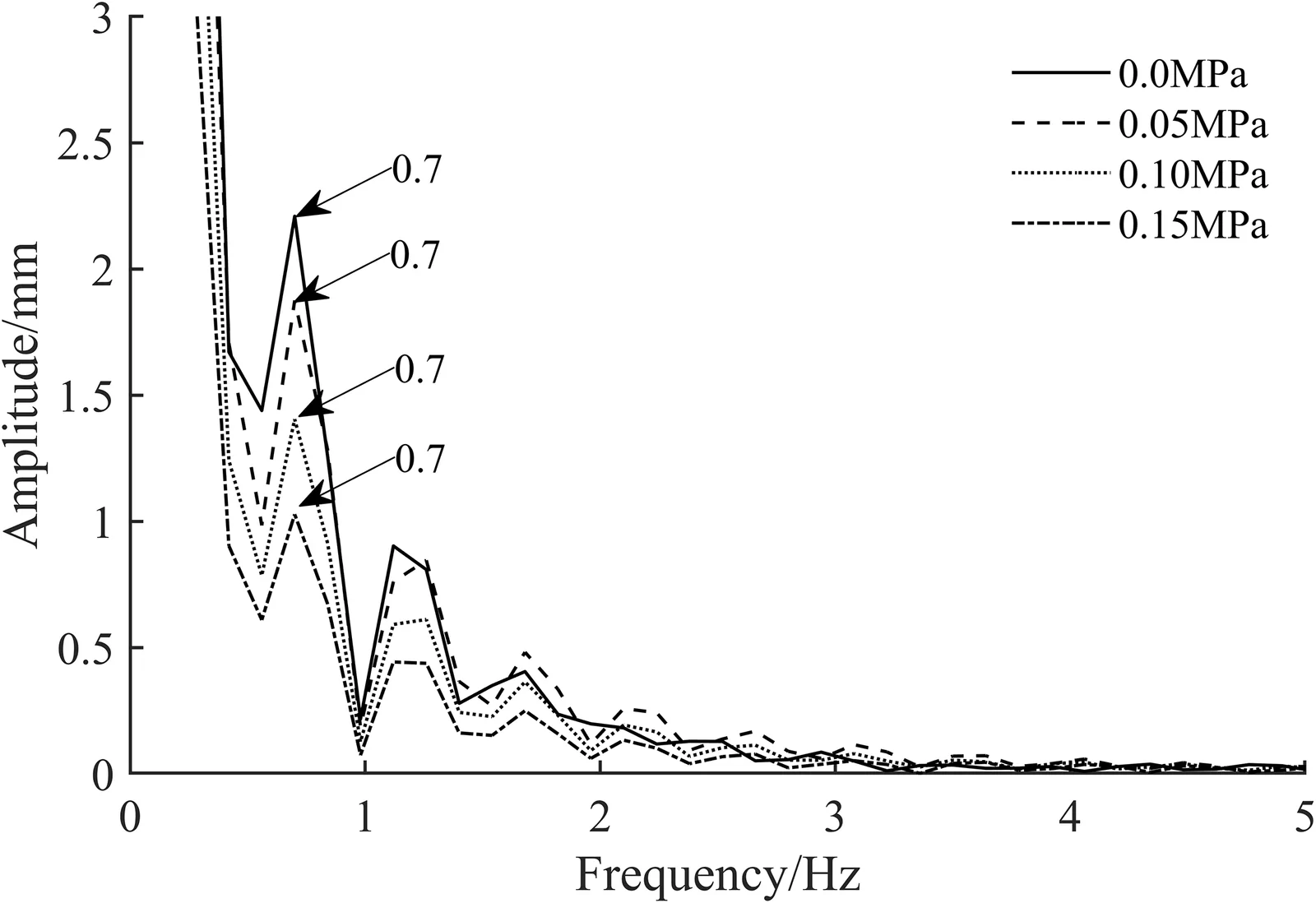
Pulse frequency-domain responses under different braking pressures.

Figs 26 and 27 show that brake activation increases system damping without changing the wrist’s natural frequency, and the vibration amplitude decreases as braking pressure increases. Relative to depressurization‑related oscillations in the non‑braked state, coordinated actuation and braking reduce angular variation and vibration amplitude during controlled pivoting. The increased damping provided by the brake is beneficial for posture maintenance; however, slight oscillations may still occur during rapid depressurization because of the elastic recovery of the pneumatic structure.

It should be noted that increasing the braking pressure can enhance system damping and reduce vibration induced by step or pulse excitations, but cannot completely eliminate it. Further vibration suppression can be achieved through strategies such as closed-loop feedback control, ramp excitation, mechanical structure optimization, and pneumatic circuit improvement. Quantitative validation of these methods is beyond the scope of this study and will be investigated in future work.

## Application-oriented validation of the flexible wrist

For the grasping experiments, the flexible wrist was connected in series with a custom five-fingered robotic hand weighing 420 g. A pneumatic control system regulated the actuation sequence and pressure during the wrist-pose and grasping experiments.

### No-load pose experiments

Adjusting the pressures in the three actuator groups enabled multidirectional pivoting about a fixed point under no-load conditions, as shown in Fig 28.

**Fig 28:**
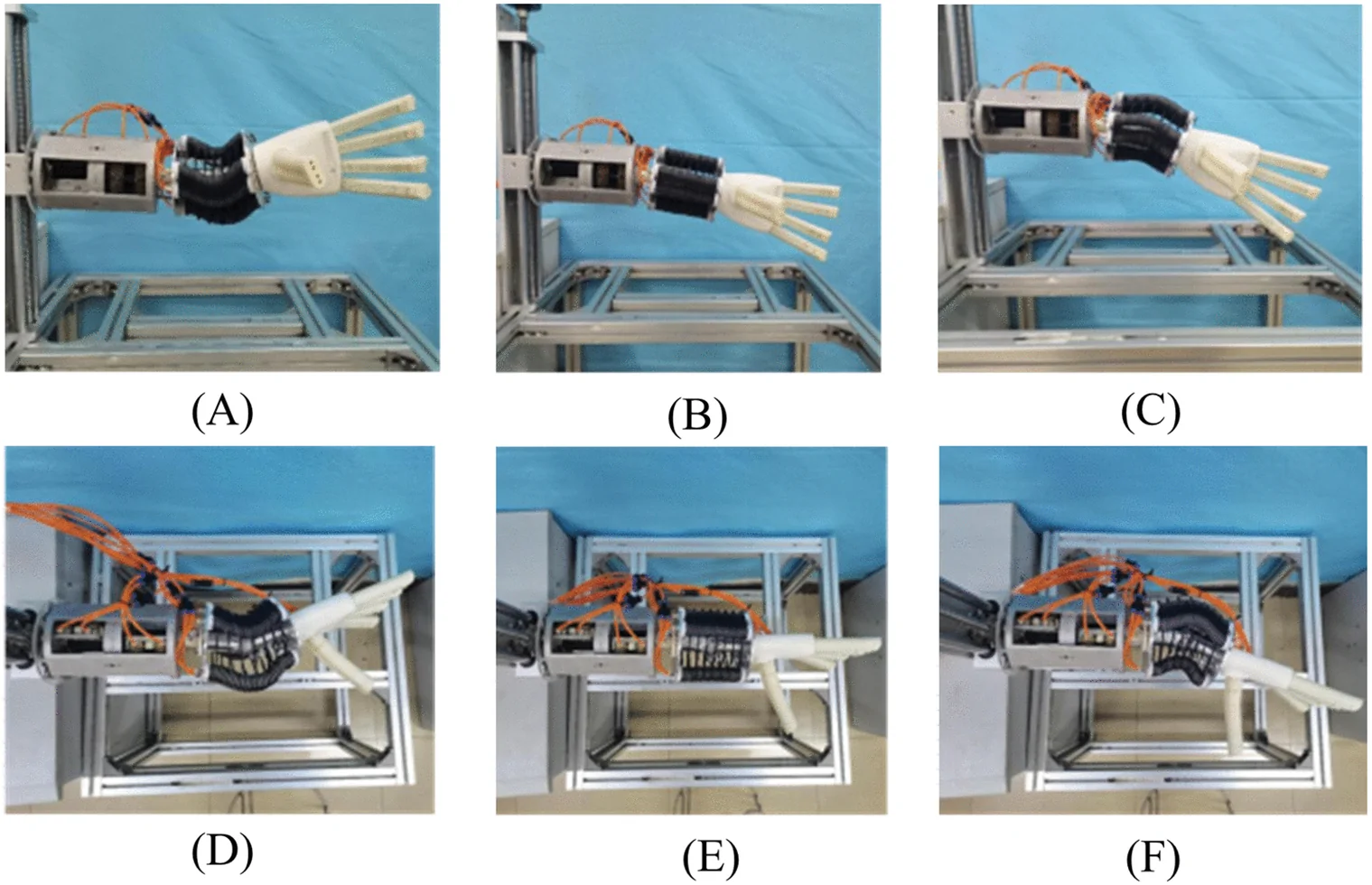
Multidirectional pivoting of the wrist. (A) Upward pitching. (B) Neutral position of pitching. (C) Downward pitching. (D) Outward yaw. (E) Yaw neutral position. (F) Inward yaw.

As shown in Fig 28B, the wrist assembly shows a noticeable downward deflection. This deflection is attributed to a pressure loss in the pitch brake, which weakens its locking action and substantially reduces bending stiffness in the pitch direction. Consequently, the weight of the robotic hand causes the wrist to deflect about the braking-ball center.

### Yaw motion of the wrist under load

Fig 29 illustrates the full four-step workflow for grasping and short-distance transportation of a 200 g mineral water bottle: (1) the sliding table moves the wrist toward the bottle and positions it for grasping, (2) the wrist brake is pressurized to maintain the posture while the robotic hand is pressurized to grasp the bottle securely, (3) the yaw-direction brake is depressurized, and (4) actuator pressure drives the wrist toward the target yaw position to complete short-distance posture adjustment and object transfer.

**Fig 29:**
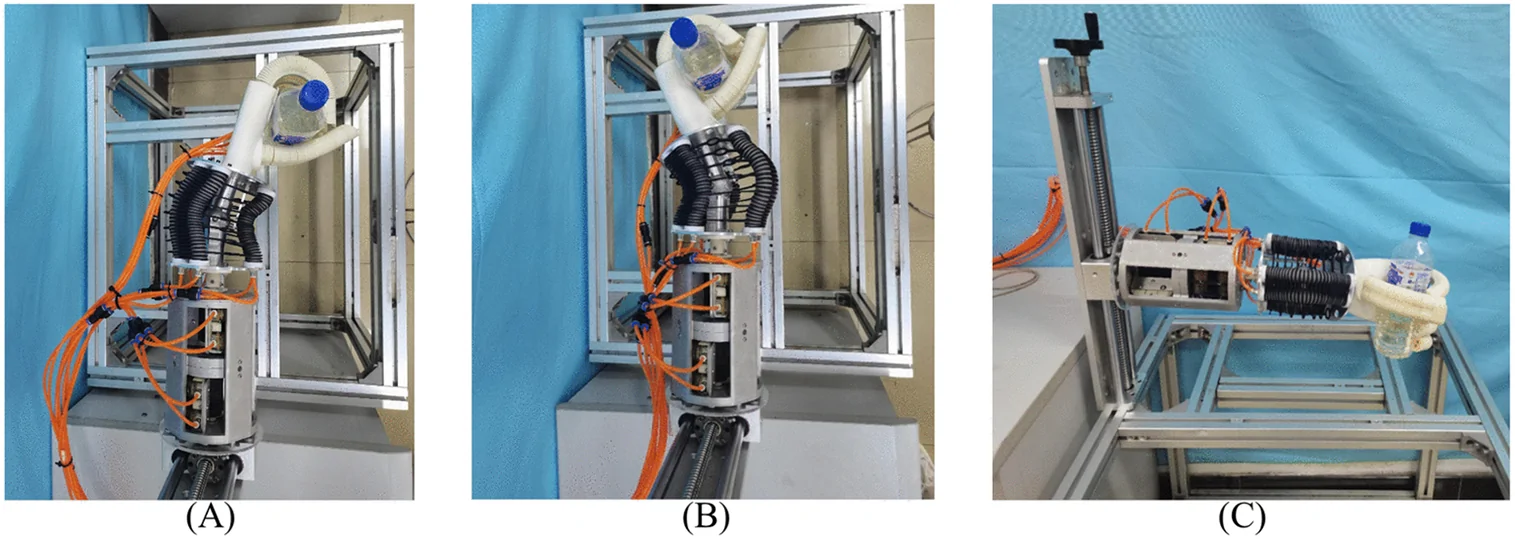
Yaw motion of the wrist under load. (A) Inward yaw. (B) Outward yaw. (C) Neutral yaw position.

In contrast to the condition shown in Fig 28B, the pitching brake in Fig 29C remains continuously pressurized during the yaw motion under load to provide stable posture-holding torque. When the wrist yaws transversely to the neutral position, the robotic hand assembly remains approximately horizontal, with no evident downward deflection. This result demonstrates that braking provides adequate structural stiffness and posture-holding capability for the tested loading condition.

### Comparative analysis with existing studies

Compared with representative pneumatic flexible wrists reported in previous studies, the primary distinguishing feature of the present prototype lies in its integrated structural scheme, in which pneumatic actuators and a pneumatic cross‑hinge brake are compactly assembled without intermediate transmission components. This architecture realizes compliant actuation together with adjustable braking torque for 2‑DOF pitch‑yaw pivoting under our experimental conditions. Assembled with a five‑fingered robotic hand, the prototype can grasp and transfer lightweight cylindrical objects for short‑range manipulation tasks in laboratory demonstrations, exhibiting preliminary application potential in biomimetic machinery, and assistive‑device scenarios.

Table 4 lists key parameters for reference. Note that direct quantitative performance comparison should be treated cautiously, since the referenced devices differ in degrees‑of‑freedom definition, structural dimension, operating pressure, and test conditions; several references also lack complete dynamic or payload data. Among the collected 2‑DOF pneumatic wrists in this table, only our design integrates an active adjustable braking mechanism. It should be emphasized that the performance metrics in the table are obtained under their respective test setups and cannot support strict one‑to‑one performance ranking.

**Table 4:** Comparison with representative existing pneumatic wrist systems.

Name	DOF	Operating air pressure	Pivot angle/°	Angle speed °/s	Braking function
Wrist rehabilitation device [36]	1	[0, 0.09] MPa	0-60	Not mentioned	No
Fully Pneumatic Robotic Wrist [11]	2	[−8, 20] KPa	±15°, ± 30	Not mentioned	No
Biomimetic Soft Robotic Wrist [27]	2	[0.068, 0.25] MPa	0-30	Not mentioned	No
Multifunctional Soft Robotic Wrist [26]	2	[0, 0.20] MPa	0-90.8	Not mentioned	No
Pneumatic Three-Finger Gripper [37]	1	[0, 0.45] MPa	0-23	Approx. 23	No
Wearable Wrist Rehabilitation Glove [38]	2	[0, 100] KPa	±50	Not mentioned	No
Flexible Wearable Wrist Power Glove [39]	2	[0, 0.10] MPa	Extension 70, Flexion 80, Radial deviation 50, Ulnar deviation 20	60	No
Wrist Brace [40]	2	[−70, 185] KPa	Extension 48, Flexion 47, Radial deviation 36, Ulnar deviation 34	Not mentioned	No
Our work	2	[0, 0.35] MPa	0-45.2	92.4	Yes; maximum braking torque: 0.842 N·m

Performance metrics from cited references are obtained under their respective experimental setups. Direct quantitative comparisons should be treated with caution due to differences in prototype dimensions, operating pressure, payload and excitation conditions across different studies.

It should be noted that the current prototype was evaluated under laboratory conditions and lightweight manipulation scenarios. Although the braking mechanism effectively improved posture maintenance and reduced vibration amplitude under controlled conditions, transient oscillations during rapid depressurization and limited braking torque under large moment-arm loading remain limitations. Future work will focus on closed-loop pneumatic control, optimized damping strategies, and enhanced brake design to improve dynamic stability and extend the applicable load range.

## Conclusions

A 2-DOF pneumatic flexible wrist with controllable braking torque was developed by combining pneumatic actuators with a cross-hinge brake. The proposed wrist allows its pivoting direction and angle to be adjusted for different operating requirements. The braking torque is also continuously adjustable to maintain the required posture. The mechanical performance of the wrist was evaluated through modeling and experiments. The main conclusions are as follows:

(1) A pneumatic actuator integrating the actuator body with the driving unit was designed and fabricated. Its elongation increased nonlinearly with air pressure, reaching 98.2 mm at 0.16 MPa, equivalent to an elongation ratio of 89.3%. The axial output force increased approximately linearly with input pressure and reached a maximum of 69.9 N at 0.30 MPa.
(2) A 2-DOF flexible wrist with controllable braking torque was developed by connecting three circumferentially distributed pneumatic actuators in parallel with a central pneumatic cross-hinge brake. Under the tested working conditions, this parallel architecture enables compliant actuation during motion and stiffness‑enhanced posture maintenance when the brake is activated. A moment-equilibrium model relating actuator pressures to the wrist pivoting direction and angle was established. The pivot-angle predictions were evaluated after empirical correction, while the pivoting-direction relationship was validated experimentally. The pivoting direction was continuously adjustable from 0° to 360° by regulating the three actuator pressures, while the maximum pivot angle was 45.2°.
(3) A pneumatic cross-hinge brake was developed, and its theoretical model was validated experimentally. The braking torque provided by the brake alone increases approximately linearly with the air pressure in the braking airbag, reaching a maximum measured value of 0.842 N·m at 0.35 MPa. Experiments on the effect of braking on wrist deformation yielded empirical equations for impedance torque and pivot angle under different operating conditions. During coordinated actuation–braking operation, the wrist’s overall resistance to external loads increased by a factor of 4.98 compared with its unpressurized initial state.
(4) The dynamic responses of the wrist under different driving pressures, braking pressures, and excitation signals were characterized using a three-dimensional motion capture system. The limiting excitation-frequency ranges for pivot angles of 15°, 30°, and 45° were identified, together with changes in natural frequency under different operating modes. Under step excitation, the natural frequency was concentrated in the low-frequency range and decreased with increasing driving pressure. Under pulse excitation, pulse width and driving pressure affected the natural frequency before the pivot angle reached steady state. Once steady state was reached, the natural frequency was independent of both factors. When the brake was engaged, braking pressure increased the overall damping and reduced the pivot angle and vibration amplitude without changing the natural frequency.

In summary, under laboratory test conditions, the developed pneumatic flexible wrist demonstrates considerable driving force and adjustable braking torque, together with relatively simple manufacturing and maintenance requirements. These characteristics indicate its potential for lightweight manipulation and assistive robotic applications, although further task-specific validation is required for applications such as agricultural picking, rehabilitation robotics, and service robots. Future work will focus on the following three aspects: (1) conducting lightweight multi-objective optimization via finite element analysis, experimental validation, topology optimization and material selection to reduce weight while preserving structural stiffness, damping characteristics and braking performance; (2) adopting dual-action artificial muscles capable of extension and contraction under variable pressurization states and implementing prestretching treatment to reduce elastic washer resistance; (3) focusing on closed‑loop pressure regulation, optimized exhaust control, and active vibration‑mitigation strategies to further improve performance for applications requiring precise posture control. These optimizations aim to increase the pivot angle to no less than 60° and a substantial improvement in load capacity, thereby providing a foundation for future development toward practical robotic applications.

## References

[1] Zhang GS, Mao ZX, Wang CB, Wei JJ. The mechanism design of a new type of wrist training and rehabilitation robot (in Chinese). Mach Des Manuf. 2022;11:268–74.

[2] Liu H, Liu Q. Study on structure design and motion characteristics of pneumatic flexible wrist with braking function. Appl Bionics Biomech. 2023;2023:1–16. doi: 10.1155/2023/2810766

[3] Ji SX, Liang YG, Hao HM, Li LN, Huang JH. Development of wrist rehabilitation device based on coaxial spherical parallel 3RRR mechanism (in Chinese). J Mech Trans. 2021;45(10):157–63. doi: 10.16578/j.issn.1004.2539.2021.10.025

[4] Azhari B, Hikmawan MF, Nugraha AS, Yazid E, Pakha AN, Baskoro CHAHB, et al. Design of switched reluctance motor as actuator in an end-effector-based wrist rehabilitation robot. J Mechatron Electr Power Veh Technol. 2024;15(2):220–9. doi: 10.55981/j.mev.2024.1109

[5] Li HL. Research on robust control strategy and joint simulation of 3-DOF hydraulic direct-drive spherical wrist [Master’s thesis]. Taiyuan University of Technology; 2024.

[6] Cheng M, Han ZN, Ding RQ, Zhang J, Xu B. Development of a redundant anthropomorphic hydraulically actuated manipulator with a roll-pitch-yaw spherical wrist. Front Mech Eng. 2021;16(4):698–710. doi: 10.1007/s11465-021-0646-2

[7] Flores OM, Lugo JH, González A, Maya M, Gonzalez-Galvan EJ, Zoppi M, et al. Design, modeling, and control of a variable stiffness device for wrist rehabilitation. Proceedings of the 2021 XXIII Robotics Mexican Congress (ComRob); Tijuana, Mexico; 2021. p. 50–55.

[8] Tanczak N, Plunkett TK, Lin S, Kuenzler L, Lau M, Kuah WKC, et al. Feasibility of post-stroke hand rehabilitation supported by a soft robotic hand orthosis in-clinic and at-home. J Neuroeng Rehabil. 2025;22(1):183. doi: 10.1186/s12984-025-01717-6 40842028 PMC12369101

[9] Zhang L, Yu Z, Su P, Li J, Ge R. Design of a parallel wrist rehabilitation robot and analysis of physiological effect on training. IEEE/ASME Trans Mechatron. 2024;29(5):3401–14. doi: 10.1109/tmech.2023.3344181

[10] Chen G, Lin T, Ding S, Chen S, Ji A, Lodewijks G. Design and test of an active pneumatic soft wrist for soft grippers. Actuators. 2022;11(11):311. doi: 10.3390/act11110311

[11] De Pascali C, Naselli GA, Palagi S, Scharff RBN, Mazzolai B. 3D-printed biomimetic artificial muscles using soft actuators that contract and elongate. Sci Robot. 2022;7(68):eabn4155. doi: 10.1126/scirobotics.abn4155 35895921

[12] Xu YJ. Design and simulation of a soft robotic device for wrist rehabilitation. Proceedings of the 3rd International Conference on Advanced Electronic Materials, Computers and Software Engineering (AEMCSE); 2020 Apr 24–26; Shenzhen, China. p. 697–701.

[13] Realmuto J, Sanger T. A robotic forearm orthosis using soft fabric-based helical actuators. Proceedings of the 2nd IEEE International Conference on Soft Robotics (Robo Soft); 2019 Apr 14–18; Washington, DC, USA. p. 591–6.

[14] Satio N, Saga N, Sato T. Development of spherical joint robot using pneumatic artifical muscles. Proceedings of the 34th Annual Conference of IEEE Industrial Electronics (IECON); 2008 Nov 10–13; Orlando, FL, USA. p. 1614–9.

[15] Li GT, Li X, Li ZQ, Tang XQ, Qin JJ. Configuration design and kinematic performance analysis of a cable-driven wrist rehabilitation robot (in Chinese). J Tsinghua Univ Sci Technol. 2026;66(02):346–56. doi: 10.16511/j.cnki.qhdxxb.2025.26.052

[16] Liao H, Chan HH, Liu G, Zhao X, Gao F, Tomizuka M, et al. Design, control, and validation of a novel cable-driven series elastic actuation system for a flexible and portable back-support exoskeleton. IEEE Trans Robot. 2024;40:2769–90. doi: 10.1109/tro.2024.3381556

[17] Jeong J, Hyeon K, Han J, Park CH, Ahn S-Y, Bok S-K, et al. Wrist assisting soft wearable robot with stretchable coolant vessel integrated SMA muscle. IEEE/ASME Trans Mechatron. 2022;27(2):1046–58. doi: 10.1109/tmech.2021.3078472

[18] Yin HB, Xue H, Zhang ZD, Li YF. Mechanism theory and motion control of humanoid wrist actuated by shape memory alloy (in Chinese). Mech Sci Technol Aerospace Eng. 2023;42(02):181–9. doi: 10.13433/j.cnki.1003-8728.20200566

[19] Nagel WS, Fakharian O, Aureli M, Leang KK. Engineered IPMC sensors: Modeling, characterization, and application towards wearable postural-tactile measurement. Smart Mater Struct. 2023;33(1):015035. doi: 10.1088/1361-665x/ad142b

[20] Zheng T, Zhang X, Wang M, Li M, Zhang C, Zhang M. Performance measurement of twisted and coiled polymer with its driven bionic wrist mechanism via temperature self-sensing. Measurement. 2025;247:116810. doi: 10.1016/j.measurement.2025.116810

[21] Zhang Y, Zhou X, Zhang F. Design, dynamic modeling, and control of a 2-DOF robotic wrist actuated by twisted and coiled actuators. IEEE/ASME Trans Mechatron. 2026;31(2):1917–27. doi: 10.1109/tmech.2025.3616174

[22] Andrikopoulos G, Nikolakopoulos G, Manesis S. Design and development of an exoskeletal wrist prototype via pneumatic artificial muscles. Meccanica. 2015;50(11):2709–30. doi: 10.1007/s11012-015-0199-8

[23] Lai J, Song A, Lu Y, Wu P, Zhang W, Li Y, et al. Design, modeling and evaluation of Lattice Structure Pneumatic Actuators (LSPAs) for soft wrist rehabilitation robot. IEEE Trans Biomed Eng. 2026;73(2):579–90. doi: 10.1109/TBME.2025.3588902 40663689

[24] Bartlett NW, Lyau V, Raiford WA, Holland D, Gafford JB, Ellis TD, et al. A soft robotic orthosis for wrist rehabilitation. J Med Device. 2015;9(3):030918. doi: 10.1115/1.4030554

[25] Al-Fahaam H, Davis S, Nefti-Meziani S. Wrist rehabilitation exoskeleton robot based on pneumatic soft actuators. Proceedings of the IEEE International Conference on Students on Applied Engineering (ICSAE); 2016 Oct 20–21; Newcastle Upon Tyne, UK. p. 491–6.

[26] Liu JP, Wang JB, Zhao W, Fei YQ. Design and modeling of multifunctional soft manipulator (in Chinese). J Mech Eng. 2022;58(09):1–9.

[27] Guan Q, Sun J, Liu Y, Wereley NM, Leng J. Characterization and nonlinear models of bending extensile/contractile pneumatic artificial muscles. Smart Mater Struct. 2021;30(2):025024. doi: 10.1088/1361-665x/abd4b0

[28] Chen XB, Zhang S, Cao KB, Wei C, Zhao W, Yao J. Development of a wearable upper limb rehabilitation robot based on reinforced soft pneumatic actuators. Chin J Mech Eng. 2022;35(04):198–206. doi: 10.1186/s10033-022-00749-6

[29] Hu T, Lu X, Yi J, Wang Y, Xu D. Biomimetic soft robotic wrist with 3-DOF motion and stiffness tunability based on ring-reinforced pneumatic actuators and a particle jamming joint. Sci China Technol Sci. 2024;67(3):774–90. doi: 10.1007/s11431-023-2579-8

[30] Liu QY, Hu P, Zhu Q, Liu T. Design and experiment of the soft driver with variable stiffness based on fiber-particle jamming (in Chinese). Robot. 2025;47(02):179–87. doi: 10.13973/j.cnki.robot.240181

[31] Zhang C, Li M, Yang Z, Kong X, Luo J, Liu Y, et al. VarWrist: An anthropomorphic soft wrist with variable stiffness. IEEE Robot Autom Lett. 2025;10(8):7883–90. doi: 10.1109/lra.2025.3579629

[32] Xia J, Cao J, Ren T, Chen Y, Chen Y, Li Y, et al. Twisting tube artificial muscle (TTAM) and Its application in agonist and antagonist drive. Biomimetics (Basel). 2026;11(1):38. doi: 10.3390/biomimetics11010038 41589956 PMC12838944

[33] Mengacci R, Keppler M, Pfanne M, Bicchi A, Ott C. Elastic structure preserving control for compliant robots driven by agonistic-antagonistic actuators (ESPaa). IEEE Robot Autom Lett. 2021;6(2):879–86. doi: 10.1109/lra.2021.3052434

[34] Zhang DH, Zhao XG, Han JD, Ma HY, Zhang B. A variable stiffness joint based on antagonistic pneumatic artificial muscles (in Chinese). Robot. 2018;40(5):587–96. doi: 10.13973/j.cnki.robot.180256

[35] Geng DX. Research on bidirectional bending active pneumatic flexible joints and its application in robot hand [Ph.D. thesis]. Jilin University; 2011.

[36] Huang L, Sun ZS, Liu YF. Wrist rehabilitation device driven by pneumatic flexible actuator. Mach Build Autom. 2016;45(06):216–9. doi: 10.19344/j.cnki.issn1671-5276.2016.06.058

[37] Bao GJ. Research on characteristics of flexible pneumatic actuator FPA and its application in multi-fingered dexterous hand design [Ph.D. thesis]. Zhejiang University of Technology; 2006.

[38] Liu YQ. Design and kinematic modeling of soft actuator for wrist movement rehabilitation [M.S. thesis]. Huazhong University of Science and Technology; 2020.

[39] Yao JT, Li HL, Cao KB, Chen XB, Zhou P, Zhao YS. Design and analysis of flexible wearable wrist power glove (in Chinese). J Mech Eng. 2019;54(19):1–9.

[40] Liu S, Fang Z, Liu J, Tang K, Luo J, Yi J, et al. A compact soft robotic wrist brace with origami actuators. Front Robot AI. 2021;8:614623. doi: 10.3389/frobt.2021.614623 33842555 PMC8027511

